# Green synthesized silver nanoparticles from *Artocarpus hirsutus* flower extract induced cytotoxicity and apoptosis in HT29 colon cancer cells

**DOI:** 10.1186/s11671-026-04834-w

**Published:** 2026-08-10

**Authors:** Shobana Sampath, Archana Hari, Blassan P. George, Douglas Law, Yashar Omarov, Aziz Eftekhari, Ayten Shukurova, Aytakin Hasanova, Ali Jimale Mohamed

**Affiliations:** 1 Department of Biotechnology, Vel Tech Rangarajan Dr. Sagunthala R&D Institute of Science and Technology, Avadi, Chennai, Tamil Nadu India; 2 Department of Biotechnology, SRM Institute of Science and Technology, Ramapuram Campus, Chennai, Tamil Nadu India; 3 Laser Research Centre, Faculty of Health Sciences, University of Johannesburg, P.O. Box 17011, Doornfontein, 2028 South Africa; 4 Faculty of Health and Life Sciences, INTI International University, 78000 Nilai, Negeri Sembilan Malaysia; 5 Faculty of Health Sciences, Shinawatra University, Pathum Thani, 12160 Thailand; 6 Department of Food Engineering and Winemaking, Azerbaijan Technological University, Ganja, Azerbaijan; 7 Department of Biochemistry, Faculty of Science, Ege University, Izmir, Türkiye; 8 Department of Pharmacognosy, Azerbaijan Medical University, Baku, Azerbaijan; 9 Department of Medical Biology and Genetics, Azerbaijan Medical University, Baku, Azerbaijan; 10 Department of Pharmacology, Faculty of Medicine, Somali National University, Mogadishu, 801 Somalia

**Keywords:** *Artocarpus hirsutus*, Silver nanoparticles (AgNPs), DNA fragmentation, Anti-apoptotic gene, Colorectal adenocarcinoma, Colorectal cancer

## Abstract

From past few years, there has been an emerging interest in environmentally friendly synthesis methods for nanoparticles due to their potential applications in catalysts, electronics, photonics, and ceramic drugs. Silver nanoparticles (AgNPs) have attention for their potential biomedical applications, including anticancer activity. This study aims to develop a sustainable approach for producing AgNPs using *Artocarpus hirsutus* as a biological source and to explore their phytochemical constituents with anticancer properties and apoptotic gene expression. The obtained AgNPs were assessed using UV–Visible spectroscopy, FTIR techniques, scanning electron microscopy (SEM), and energy-dispersive X-ray (EDAX) analysis. SEM examinations demonstrated that the produced AgNPs predominantly displayed both spherical and irregular morphologies, with an average dimension falling within the 20 to 50 nm range. FTIR analysis results indicated the availability of biological compounds in the methanol flower extract of *Artocarpus hirsutus* which function as reducing and capping agents for the bio fabrication of AgNPs. To assess their cytotoxic effects, experiments were conducted on human colon cancer HT-29 cells in vitro through DNA fragmentation, DCFDA staining, flow cytometry, and MTT assays. Different doses (25, 50, 100, 250, and 500 µg/ml) of the methanol flower extract were exposed to HT-29 colorectal adenocarcinoma cells, and the synthesized AgNPs exhibited dose-dependent cytotoxicity, promoting apoptosis in the cell lines. Quantitative RT-PCR was used to confirm the Significant down-regulation of anti-apoptotic Bcl2 in HT-29 colorectal adenocarcinoma cells treated with synthesized silver nanoparticles (AgNPs). The findings indicate that the green synthesized AgNPs exhibit significant cytotoxic and apoptosis-inducing effects in HT-29 colorectal cancer cells. However, further studies involving normal cell lines and in vivo models are required to validate their therapeutic potential.

**Supplementary Information:**

The online version contains supplementary material available at 10.1186/s11671-026-04834-w.

## Introduction

Over the past few years, cancer has become the second leading cause of fatalities and resulted in an estimated 9.6 million fatalities on a global scale in the year 2018 [1]. Cancer became the most prominent life-threatening burden worldwide, causing proliferations of abnormal cells, and some of the prevalent types of cancers are breast, liver, lung, prostate and colorectal cancers. The World Health Organization foresees a significant increase in both the incidence and mortality of cancer globally by 2040. There have been 27.5 million reported instances of the condition, resulting in 16.3 million fatalities, primarily driven by population growth and aging [2]. Globally, colorectal cancer leads the third most common cause of cancer and is characterized as a carcinoma that impacts the inner lining of the colon’s mucosal membrane. Although various therapeutic approaches, such as surgical procedures, chemotherapy, and radiological treatments, are employed to treat colon cancer patients, these methods are highly expensive, often inaccessible, and can cause adverse effects on healthy cells [3–5]. Therefore, there is a pressing necessity to discover an alternative approach to cancer treatment, with nanotechnology emerging as a novel frontier that utilizes nanoparticles for both onco-diagnosis and therapeutic applications.

Nanotechnology is the branch of applied science as the field ranges from matter to the molecular level at the nanoscale, generally 1 to 100 nm, and the fabrication of devices within this size range [6]. Nanotechnology, a term coined by Japanese researcher Norio Taniguchi in 1974 refers to substances, called nanoparticles with at least one external dimension of 100 nm or less, as defined by ISO [7].Nanoparticles have a wide range of utilizations in the electronic appliances, transit, telecoms, bio-imaging, health care, environmental cleanup, beauty products, and surface protective coatings [8–10]. Metallic nanoparticles are synthesized using versatile approaches and hold substantial significance in biomedicine, particularly in areas such as antimicrobial applications, antitumor therapies, targeted drug and gene delivery, cellular visualization, food packaging, and biosensors, etc. [11–14]. The biological synthesis of metallic nanoparticles has become increasingly popular as a feasible substitute for traditional methods. This is mainly because it is simple, fast, safe, non-toxic, cost-effective, and environmentally friendly. This technique surpasses the challenges typically linked with physical and chemical methods. Hence, metallic nanoparticles are safe to produce from biological systems such as fungi, bacteria, yeasts, plant extracts, and actinomycetes at the extracellular and intercellular levels [15–18]. Emerging evidence suggests that plant-mediated silver nanoparticles possess strong anticancer potential by inducing apoptosis through multiple mechanisms. Studies using flow cytometry have confirmed significant increases in early and late apoptotic cell populations, indicating effective activation of programmed cell death pathways. These effects are often associated with reactive oxygen species (ROS) generation and mitochondrial dysfunction, highlighting the therapeutic relevance of such nanomaterials [19].

Recently silver nanoparticles (AgNPs) have major applications in the field of medicine and in consumer products [20–22].Green synthesis of silver nanoparticles using plant extracts has gained significant attention due to its eco-friendly nature and enhanced biological activity. Such biosynthesized nanoparticles have been reported to exhibit antimicrobial and anticancer properties, making them promising candidates for biomedical applications [23]. They have become increasingly prevalent in a wide range of applications, spanning from food, pharmaceuticals, and everyday consumer goods to household appliances. Notably, their use has gained momentum in the field of cancer diagnosis and treatment. The present synthesis method is unique in utilizing *Artocarpus hirsutus* flower extract as both a reducing and stabilizing agent, eliminating the need for additional chemical reagents. This simple and cost-effective approach enables the formation of stable AgNPs with controlled size and morphology. AgNPs are not only valuable for delivering precise drug delivery and acting as probes for cancer detection but also hold promise as a potential standalone therapeutic agent in cancer therapy. AgNPs also have anti-angiogenic properties which have biological application to inhibit angiogenesis [24]. The research shows that synthesized silver nanoparticles (AgNPs) through a biogenic process leads to the creation of new bioactive nanomaterials with extraordinary potential for application in medicine. It has been shown that these biosynthesized nanoparticles will cause cytotoxicity in a concentration-dependent manner, as well as cause apoptosis (cell death), as evidenced by increased populations of cells in both the early and late stages of apoptosis. These unique biological properties offer new opportunities for using AgNPs in other treatment options for cancer [25]. Silver nanoparticles (AgNPs) have gained a lot of attention as potential cancer-fighting agents and have been found to have a toxic effect on different cell lines, including Hep2, A549, HepG2, HT29, HELA, Caco_2_, MCF-7, and HCT-116 colon [26–28]. AgNPs work mainly by promoting cell death through various processes such as generating reactive oxygen species (ROS), inducing cellular apoptosis, and causing fragmentation of DNA. The report indicated that, silver nanoparticles (AgNPs) demonstrated significant toxicity towards SW480 human colon cancer cells, but did not show any harmful effects on healthy peripheral lymphocytes (PLs) [29]. Our interest in cancer studies on discovering various plant species for the purpose of green synthesis of AgNPs has led us to utilize an extract from the flower of *Artocarpus hirsutus* in our recent and distinctive attempt to synthesize silver nanoparticles.

*Artocarpus hirsutus*, which belongs to the *Moraceae family,* comprises 60 genera and over 1000 species [30]. This species can be found across the tropical and subtropical regions of Asia, spanning a broad geographical area and is useful as food and traditional medicine. *Artocarpus hirsutus* also known as wild jack is an endemic tree species which are found in the Western Ghats of India [31]. Different parts of the *Artocarpus* species have great medicinal values in Ayurvedic, Siddha, and Unani. The roots and barks are used to treat diarrhea; leaves with white camphor are used to cure venereal buboes [25]. This property has created interest to study the anticancer nature of silver nanoparticles produced by the *Artocarpus hirsutus* flower extracts. The in vitro studies on the AgNPs from the wild jack flower extracts have exhibited significant cytotoxic profile on various cancer cells such as breast, ovary, brain, cervix etc., This toxic profile of the nanoparticles may be due to the disruption of mitochondrial chain, production of ROS and also interrupting the production of ATP synthesis [32].

This research study presents a synthesis of silver nanoparticles using *Artocarpus hirsutus* flower extract, which can act as a reductant and stabilizer, and provides the opportunity to test the effectiveness of the nanoparticles against HT-29 cells. The present study uses *Artocarpus hirsutus* because of its high levels of bioactive phytochemicals, namely phenolic compounds and flavonoids, which may facilitate the synthesis of silver nanoparticles and enhance their effectiveness. Unlike previous studies that simply described the process of nanoparticle synthesis or their general cytotoxic characteristics, the present study performed phytochemical analysis, nanoparticle characterization, ROS generation, and DNA fragmentation analysis in addition to studying the activity of green-synthesized AgNPs.

This study investigates the environmentally friendly production of AgNPs using the *Artocarpus hirsutus* flower extracts. The AgNPs produced through this biogenic approach were subject to various characterization techniques, including UV–Vis spectroscopic analysis, scanning electron microscopy (SEM), Fourier transform infrared (FTIR) techniques, and energy-dispersive X-ray (EDAX) analysis. This study presents the report on the in vitro anticancer property of the green synthesized silver nanoparticles through various analysis MTT assay, gene expression profile, and DNA fragmentation and also by staining assays in the HT29 colorectal adenocarcinoma cell lines. In conclusion, the synthesized Ag-NPs utilizing an extract of *Artocarpus hirsutus* flower and characterizing the cytotoxicity and apoptosis-inducing activity of the synthesized Ag-NPs in HT-29 colonic cancer cell lines and their possible mechanisms of action involving the generation of ROS and modulation of apoptotic gene expression in vitro only at present, with need for future in vivo studies to determine clinical effects.

## Materials and methods

### Materials

Freshly dried *Artocarpus hirsutus* flower were appropriately identified and procured from the medicinal market in the Nagercoil district, Tamil Nadu, India. All reagents including C_2_H_3_AgO_2_ (silver acetate), NaOH, H_2_SO_4_, FeCl_3_, phosphate buffer and ethidium bromide, ethyl alcohol, Dulbecco's Modified Eagle Medium (DMEM), ninhydrin solution, Benedict’s reagent, potassium ferric cyanide, potato dextrose agar (PDA), Dimethyl sulfoxide (DMSO), and trichloroacetic acid (TCA), were purchased from India and used without further purification. The cell lines of human colon cancer (HT29) were procured from the National Center for Cell Sciences (NCCS), Pune, India.

### Preparation of flower extract

The blossoms of *Artocarpus hirsutus* were gathered by carefully eliminating the calyx and pollen grains. Subsequently, the flowers were exposed to a period of seven days of shade drying to ensure thorough moisture removal, thus preventing the growth of fungi and the degradation of heat-sensitive compounds. Extract of *Artocarpus hirsutus* was prepared by boiling 10 g of grounded wild jack flowers in 100 ml of hexane, ethyl ethanoate, and methanol and further filtered using Whatman filter paper. These solutions are cooled to room temperature and then used for the further investigations.

### Phytoconstituents analysis

Phytochemical screening of the raw extract of *Artocarpus hirsutus* flowers was investigated using a number of solvents, including hexane, ethyl acetate and methanol. The following phytoconstituents were examined with phenolic compounds, terpenoids, saponins, glycosides, flavonoids, tannins, resins, alkaloids, sugar reduction, and steroids. However, quantitative testing was pursued for the assessment of phenol and flavonoid compounds [33].

### Antioxidant activity of* Artocarpus hirsutus* extract

#### Dot-blot assay

*Artocarpus hirsutus* pipette (3 μl) was spotted on a Thin-Layer chromatographic (TLC) plate for various solvent extracts. The TLC plate carrying with dry spots were turned upside down and left in that position for 10 s. Further they were permitted to settle for few min, then added to the 1, 1-diphenyl 2-picrylhydrazile (DPPH) solution. The spots that exhibit radical scavenging activity, such as yellow spots appearing in the purple extract, indicate the presence of antioxidants [34].

#### Radical scavenging assay

The antioxidant efficacy of the raw flower extracts of *Artocarpus hirsutus* were evaluated on the premise of stable free radical scavenging property. Sample solutions made up of each solvent extracts (1:1 mg/mL) at varying conc. of 100 μg/mL to 500 μg/mL, etc., for each from a test tube. The mixture made up of 1 mL of Dimethyl Sulfoxide (DMSO) and 1 mL of 1,2 di-phenyl picryl hydroxyl (DPPH- 0.1 mM) solution was added. Finally, sustained at 30 min of dark incubation, the absorption value was estimated at 517 nm using the UV–Vis spec [35].

#### Total antioxidant capacity (Phosphomolybdenum Assay)

Multiple pipetting *Artocarpus hirsutus* crude samples were administered (1:1 mg/mL), at different concentrations (200, 400, 600 to 1000 μg/mL) etc. Next, a 1 mL aliquot of the stock solution was prepared and added to each of the extracts. The test tubes were then carefully sealed and incubated in a water bath at 95 °C for duration of 90 min. After this incubation period, the samples were allowed to cool down to 25 °C, and the absorbance was subsequently measured at a wavelength of 695 nm [36].

#### Reducing power assay

Various concentrations of *Artocarpus hirsutus* of methanol extract (200, 400 600 to 1000 μg / mL, etc.) were taken, and the corresponding concentrations were combined with potassium ferricyanide (2.5 mL) and phosphate buffer (2.5 mL). The solution was placed at water bath for a period of 25 min at 50^o^ C, while cooling down, 3.0 mL 10% of trichloroacetic acid has been introduced and vortexes for 3500 rpm for a period of 15 min. The uppermost layer of the solution was separated, and after that, it was mixed with 3.0 mL of distilled water and a newly prepared solution of ferric chloride (1.0 mL). Subsequently, the absorbance was measured at a wavelength of 750 nm, using 1 mL of methanol as a reference blank [37].

#### Lipid peroxidation assay

A 10% w/v homogenate of goat liver was suspended in a 0.1 M phosphate buffer with a pH of 7, and then it was subjected to centrifugation at 8000 rpm for 10 min (aqueous phase has been collected). 0.5 mL sample was subsequently extracted from the top layer and placed into each test tube, with each tube containing varying concentrations of the extracts of *Artocarpus hirsutus.* The entire volume consisted of 1 mL, which was composed of distilled water. Then FeSO_4_ was applied to each test tube, incubated at 37 ^o^C for 30 min. Solution A was however prepared, (Solution A- taken 20% acetic acid, 0.8% TBA (Thiobarbituric acid) in 1.1% SDS) added to 1.5 mL in each test tube, and then added TCA to all test tubes (1.5 mL), kept for incubation with a period of 60 min at 95 °C. After the incubation period, 5 mL of butanol were introduced into each tube and then subjected to centrifugation at 3000 rpm for duration of 10 min. This had been estimated to take the upper layer of solution, absorbance at 532 nm [38].

### Biosynthesis of silver nanoparticles

Silver nanoparticles were synthesized using plant extract under controlled conditions. Briefly, 0.1 M silver acetate solution was mixed with 30 mL of extract (1:1, v/v; final concentration of 0.05M) and incubated in dark conditions overnight. The resulting nanoparticles were collected by centrifugation at 9000 rpm for 30 min, followed by washing with acetone and drying [39]. Afterward, the nanoparticle pellet was suspended in acetone and dried.

### Characterization of silver nanoparticles

The green-synthesized silver nanoparticles (AgNPs) have been subjected to various characterization techniques. Initially, they were diluted and analyzed using UV–Visible spectroscopy. The UV–Vis spectra of the specimens were measured using a UV-1700 Pharma spectrometer, covering wavelengths from 200 to 800 nm at a 1 nm resolution. Distilled water was used as a reference control in this analysis. To identify the potential functional groups in the biomolecules accountable for the lowering and capping of the AgNPs, Fourier Transform Infrared (FTIR) spectroscopy was employed. The FTIR measurements were conducted using an RX1-Perkin Elmer spectrophotometer over a wavenumber range of 4000–400 cm^−1^ to identify the functional groups involved in the reduction and stabilization of the synthesized silver nanoparticles. The crystalline structures of the *A. hirsutus* AgNPs sample were examined using X-ray diffraction. The analysis was conducted using a PANalytical X'pert X-ray diffractometer, which was operated at 45 kV with a current of 30 mA, and utilizing of the Cu-Kα radiation. The XRD analysis covered a scanning range from 10 to 90 degrees at a 2θ angle. For the assessment of the dimensions and structure of the AgNPs, a sample was prepared for scanning electron microscopy (SEM). The procedure involved drop-coating the biologically synthesized AgNPs solution onto a copper (Cu) grid coated with carbon and then eliminating any surplus solution using blotting paper. Subsequently, the SEM grid was allowed to dry for 5 min before analysis. To perform a quantitative elemental analysis of the silver nanoparticles, energy dispersive X-ray absorption spectroscopy (EDAX) was employed.

### Cytotoxicity assay

Cytotoxicity of the synthesized silver (AgNPs) nanoparticles was studied against HT-29 colon cancer cell lines using MTT cell viability assay where the colorectal adenocarcinoma cell line (HT29) were individually seeded in 96-well plates at a density of 1 × 10^4^ cells/well using DMEM media, further the cell lines were processed with different experimental concentration (25–500 μg/ml) of samples in serum free media for an incubation period of 24 h. Cells were seeded at a density of 1 × 10^4^ cells/well and incubated for 24 h. After treatment, cell viability was assessed using MTT assay, and absorbance was measured at 570 nm. The assessment of the growth of color intensity was conducted using a wavelength of 570 nm. Cell viability was measured by MTT assay, to determine the concentration at which the cytotoxicity has been assessed [40, 41]. The concentration range of 25, 50, 100, 250, and 500 μg/mL was selected based on previously published studies evaluating the dose-dependent cytotoxic effects of green-synthesized silver nanoparticles against human cancer cell lines. This concentration range was considered appropriate to evaluate both low and high dose cellular responses, determine the IC₅₀ value, and facilitate comparison with similar studies reported in the literature [42].

### DCFDA staining

The research assessed the production of reactive oxygen species (ROS) within HT29 colon cancer cell lines due to the application of produced silver nanoparticles with the fluorescence marker H_2_DCF-DA (2, 7-dichlorofluorescein diacetate) staining (Miethling-graff et al. 2014). About 6 X 10^4^ cells were placed individually in the well of 12-well plates and given time to adhere overnight. The nanoparticles were added and incubated for 8 h at their IC_50_ values. After the drug treatment, the media was taken out, and the cells were exposed to a solution containing 5 mM H2DCF-DA, which was diluted in clear media, for duration of 30 min at a temperature of 37 degrees Celsius [43]. H_2_O_2_ was used as a positive control. The cells were rinsed thrice with PBS, and their appearance was captured under a Nikon ECLIPSE TE2000-U microscope (Nikon Instruments Inc., Melville, NY, USA) with an excitation wavelength of 485 nm and emission at 530 nm.

### Apoptotic DNA laddering analysis

Laddering assay is widely employed to detect the programmed cell death which also analyses the inter-nucleosomal cleavage of DNA. HT29 colon cancer cell lines were employed under the IC_50_ concentration on AgNPs for 24 h. After Following the incubation period, the cells that had undergone treatment were collected and rinsed using phosphate-buffered saline (PBS). Subsequently, they were subjected to lysis by using 500 µl of a lysis buffer under mild agitation at 55 °C for duration of 2 h, ensuring the complete digestion of any solid material. The DNA was then isolated through extraction with a mixture of phenol and chloroform in a 24:1 ratio. Then it was subjected to ethyl alcohol precipitation, leading to the formation of a DNA pellet. This pellet was subsequently air-dried. The DNA pellet was then reconstituted in a 20 µl solution of TE buffer, which contains 50 µg/ml of RNase-A, followed by a 30 min incubation at 37 °C. After this, the sample was examined for DNA laddering analysis through electrophoresis on an agarose gel. Finally, the gels were Stained with Eco dye nucleic acid stain and visualized under UV transilluminator [44].

### Flow cytometry analysis

Flow cytometry was used to determine the distribution of cells in different stages of the cell cycle and to calculate the percentage of cells undergoing apoptosis. Summarily, HT-29 carcinoma cell lines that had been cultured were collected through the process of trypsinization; the experiment involved seeding cells at a concentration of 2 × 10^4^ cells in a 6 well tissue culturing plate, using DMEM medium with 10% FBS and 1% antibiotic solution. The cells were then incubated for 24–48 h at 37 °C. Subsequently, the wells were rinsed with sterile PBS and exposed to silver nanoparticles (AgNPs) at their IC_50_ concentration, using serum-free DMEM medium. This mixture was then incubated at 37 °C in a 5% CO_2_ incubator for 24 h. After this incubation period, the cells were collected and fixed with cold 70% ethanol for 30 min at 4 °C. Following fixation, the cells were washed with sterile PBS, and the cell pellet was treated with 50 µl of RNase (100 µg/ml) and 500 µl of PI (100 µg/ml) before being stored at 4 °C. Finally, the cell samples were analyzed using a flow cytometer [45].

### Protein expression profiling

The study aimed to explore the produced nanocomposites that affect the anti-apoptotic protein Bcl-2 in HT29 colorectal carcinoma cell lines, a protein crucial for preventing cell death through both mitochondrial and death receptor pathway. To conduct the experiment, HT-29 cells, a human colorectal adenocarcinoma cell line, were cultured in DMEM medium supplemented with 10% FBS, 100 u/ml penicillin, and 100 µg/ml streptomycin. The cells were maintained in an environment with 5% CO_2_ at 37 °C. The cultured HT29 colorectal adenocarcinoma cells were harvested through trypsinization and collected in a 15 ml tube. Subsequently, these cells were placed in 6-well tissue culture plates at a density of 1 × 107 cells/ml (1 mL per well) in DMEM medium containing 10% FBS and a 1% antibiotic solution [46]. They were allowed to incubate for 24 h at 37 °C. Following this incubation period, the wells were rinsed with sterile PBS and then separately treated with silver nanoparticles (AgNPs) at their respective IC_50_ values in a serum-free DMEM medium. The cells were then incubated at 37 °C in a humidified 5% CO_2_ incubator for another 24 h. After this incubation, all HT29 carcinoma cells were collected via trypsinization and gathered in a centrifuge tube for further analysis.

The RNA extraction from HT29 colorectal carcinoma cell lines involved using the TRIZOL method. Initially, around 10 million cells were spun down in centrifuge tubes treated with DEPC at 5000 rpm for 10 min, forming a cell pellet. This pellet was then subjected to cell lysis by adding 700 µl of TRIZOL. After lysis, the solution was moved to 1.5 ml tubes, and 300 µl of chloroform was introduced, followed by vigorous mixing for 5 min at room temperature. The centrifugation process was used to separate the aqueous layer, which was then collected in a fresh 1.5 ml tube. Isopropanol was added to precipitate the RNA, and the resulting RNA precipitate was gathered through centrifugation. Finally, the RNA pellet was air-dried and dissolved in 30 µl of double-distilled autoclaved water, and it was stored at − 80 °C. The Labman UV–Vis Spectrometer was employed to evaluate both the quantity and quality of the isolated RNA, and the RNA was examined in a 1.5% agarose gel. For the gene-level detection of micro-metastases, 1.5 µg of total RNA was transformed into cDNA using a reaction mixture that included reverse transcriptase (MMLV). The cDNA synthesis took place at 25 °C for 10 min, followed by 37 °C for 120 min. To complete the process, the cDNA and RNA hybrid was denatured, and reverse transcriptase was inactivated at 85 °C for 2 min [47].

The cDNA we generated was utilized as a template for detecting metastasis. We employed qRT-PCR with the Power Syber Green kit from Applied Biosystems, CA, USA, in the ABI Step One Plus instrument, also from Applied Biosystems, CA, USA. To assess the expression levels of specific genes (refer to Primer sequence Table 1), we employed the relative quantification method (2^ΔCT^). The expression data were normalized using Beta-actin as the endogenous control, with clinically normal cells serving as the reference or calibrator.

**Table 1:** Primer sequences of *Bcl-2* and *β*-*actin* genes used in the real time PCR assay

Apoptosis regulator	Direction	Sequence (5´–3´)
Human β-Actin	Forward	5' CACCAACTGGGACGACAT 3'
	Reverse	5' ACAGCCTGGATAGCAACG 3'
Human B-cell Lymphoma 2	Forward	5'-CCTGTGGATGACTGAGTACC-3'
	Reverse	5'-GAGACAGCCAGGAGAAATCA-3'

### Statistical analysis

The data was plotted using GraphPad Prism 9, and the experiments were conducted three times. The mean values along with standard deviations were reported as means ± S.D. The statistical significance of the results was assessed through a one-way analysis of variance using IBM SPSS statistical software, with a significance level set at *P* < 0.05. The software ED50PLUS V1.0 was utilized to compute the LC50 value for cytotoxic analysis.

## Results and discussion

### Extraction of *A. hirsutus* blossoms

**Fig. 1:**
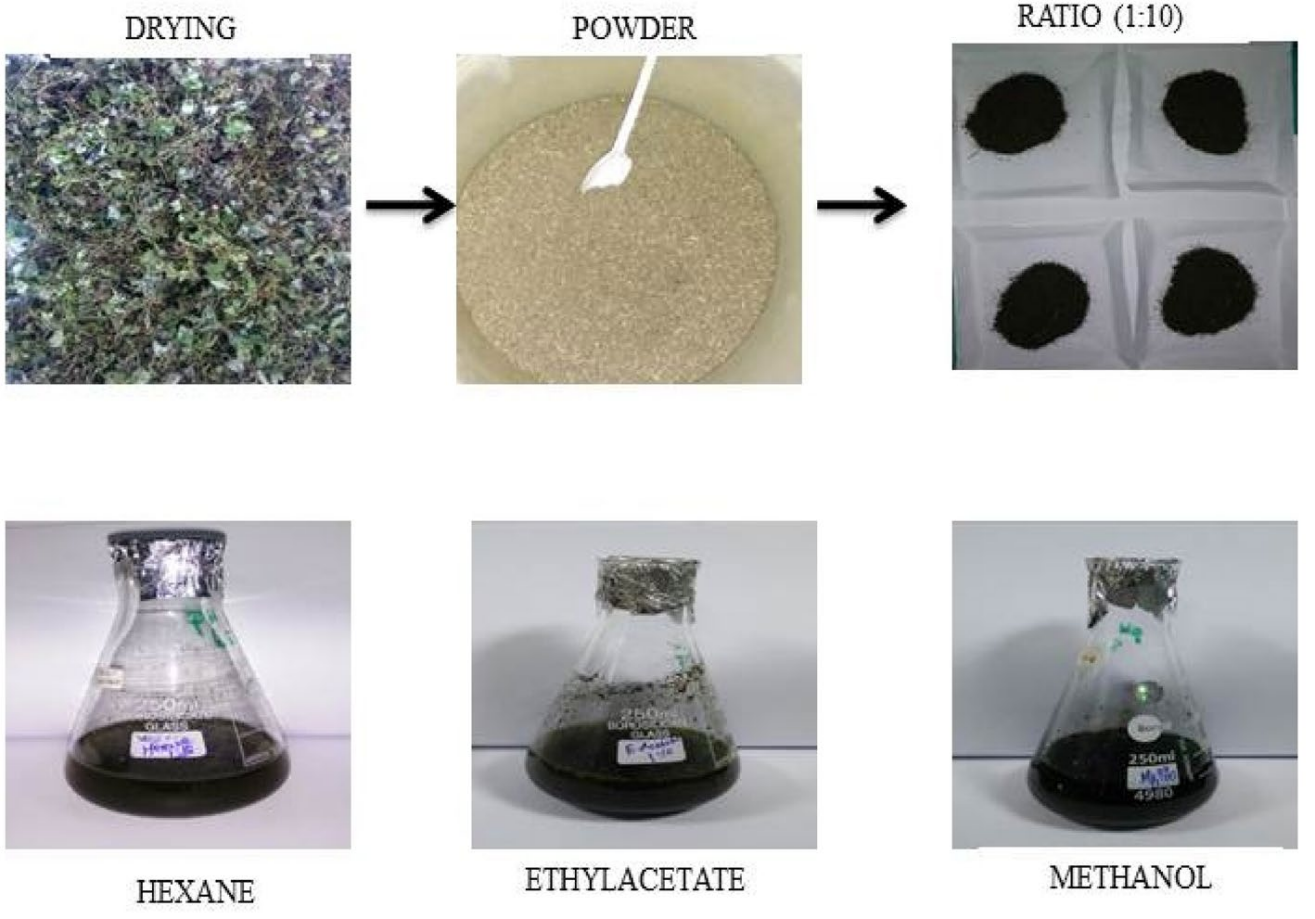
Extraction of *Artocarpus hirsutus* flower extract

Preparation for the extraction of *Artocarpus hirsutus* blossoms was performed using a number of solvents, including hexane, ethyl ethanoate and methyl alcohol. The extraction method was carried out by drying the plant material into a molten powder at 37 °C, finally taking the polarity-based solution with (1:10) solvents and are shown in Fig. 1.

### Phytoconstituents analysis

The screening tests showed that the different solvent extracts of *Artocarpus hirsutus* had phenols, flavonoids, alkaloids, terpenoids, tannins, saponins, carbohydrates, tannins, glycosides, resins and steroids etc., The phytochemical screening results of *Artocarpus hirsutus* are tabulated in Table 2. All these phytoconstituents were extensively present in methanol solvent extract compared to ethyl acetate and hexane extract. Compounds such as alkaloids and saponins have not been detected in the extract of ethyl acetate. Phenol, alkaloids, saponins, tannins and resins were absent from hexane extract. The predominant phytochemicals, such as phenols and flavonoids, were further quantified. Quantitative analysis of the methanol extract of Artocarpus was conducted using aluminium chloride tests to detect the amount of flavonoid. The findings showed that flavonoid was present in a large amount relative to other constituents and relative to regular quercetin [48]. The findings indicate that the quantity of flavonoids in the extract was 1234.94 quercetin equivalents (QE) and the phenolic content was 79.99 gallic acid equivalents (GAE) compared to gallic acid. Flavonoid primarily contributes to antioxidant and free radical scavenging properties that are useful for the exploitation of their pharmacological activities (e.g. anti-diabetic, anti-cancer, anti-microbial).

**Table 2:** Phytochemical constituents from the flower extract of *Artocarpus hirsutus*

S.No	Phytochemical constituents	Test	Result		
			Hexane	Ethyl -acetate	Methanol
1	Phenol	Ferric Chloride	−	+	+
2	Flavonoids	NaOH	+	+	+
3	Saponins	Foam Test	−	−	+
4	Steroids	Salkowski Test	+	+	+
5	Terpenoids	Salkowski Test	+	+	+
6	Reducing sugar	Fehling's Test	−	+	+
7	Tannins	Ferric Chloride	−	+	+
8	Glycosides	Keller Test	+	+	+
9	Alkaloids	Mayer’s Test	−	−	+
10	Resins	Cu-acetate Test	−	+	+

### Antioxidant assay of *A. hirsutus* flower extract

#### Dot-blot assay

**Fig. 2:**
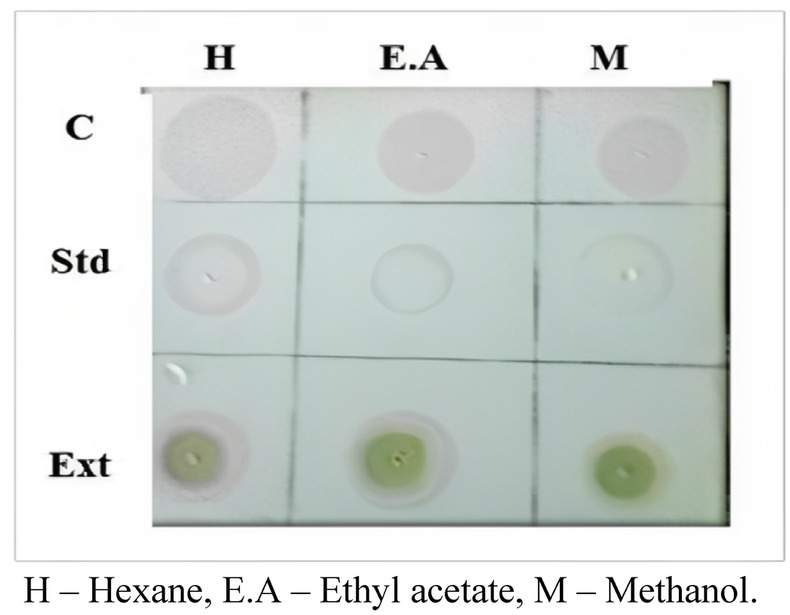
Dot-Blot Assay of *Artocarpus hirsutus* flower extract

The outcome shows that the Dot-Blot assay's preliminary screening of scavenging activity concludes that the yellow color ring developed in the context of various extracts of *Artocarpus hirsutus* and is shown in Fig. 2.

Usually, this approach focuses on preventing the accumulation of oxidized compounds, as the introduction of antioxidants prevents the production of free radicals. The appearance of a yellow spot against a purple background is of great screening significance for the assessment of the antioxidant potential of the plant extract [49].

#### Radical scavenging activity

**Fig. 3:**
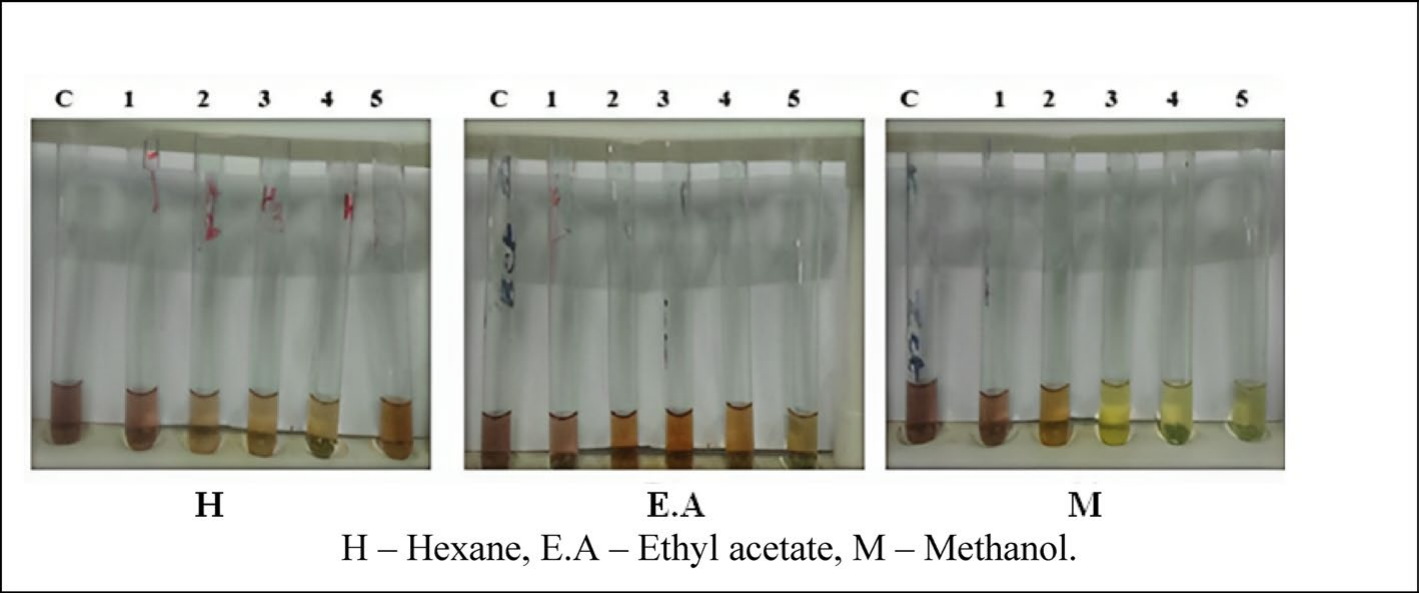
DPPH activity of *Artocarpus hirsutus* flower extract

**Fig. 4:**
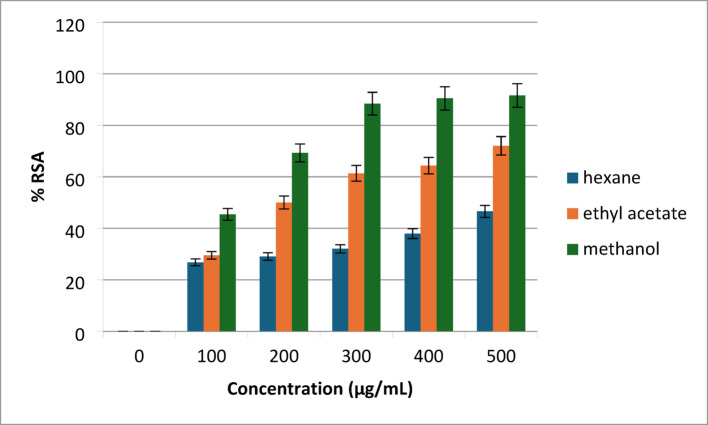
% Radical scavenging activity (RSA) of *Artocarpus hirsutus* flower extract

DPPH radical scavenging assay is a commonly employed method to assess and screen antioxidant properties of crude extracts. The antioxidant activity of hexane, ethyl acetate and methanol extracts of *Artocarpus hirsutus* are illustrated in Fig. 3. The IC_50_ values for hexane, ethyl acetate and methanol extracts were 536.59 μg/mL, 199.64 μg/mL and 144 μg/mL, respectively. Based on the results obtained, the extract of methanol had substantial radical scavenging ability compared to the extracts of hexane and ethyl acetate, as shown in Fig. 4. DPPH experiments have been widely used to analyze antioxidant Flower extracts function as free radical scavengers or hydrogen donors [50, 51]. The primary scavenging activity of *Artocarpus hirsutus* was attributed to the presence of phenols and flavonoids [52]. DPPH activity in plant extracts of *Artocarpus hirsutus* may also be partly due to Millard reaction products rather than phenolic components, as they are also involved as radical scavengers [53]. The results obtained were close to those reports investigated earlier by Thamacin et al. [54].

#### Phosphomolybdenum assay

**Fig. 5:**
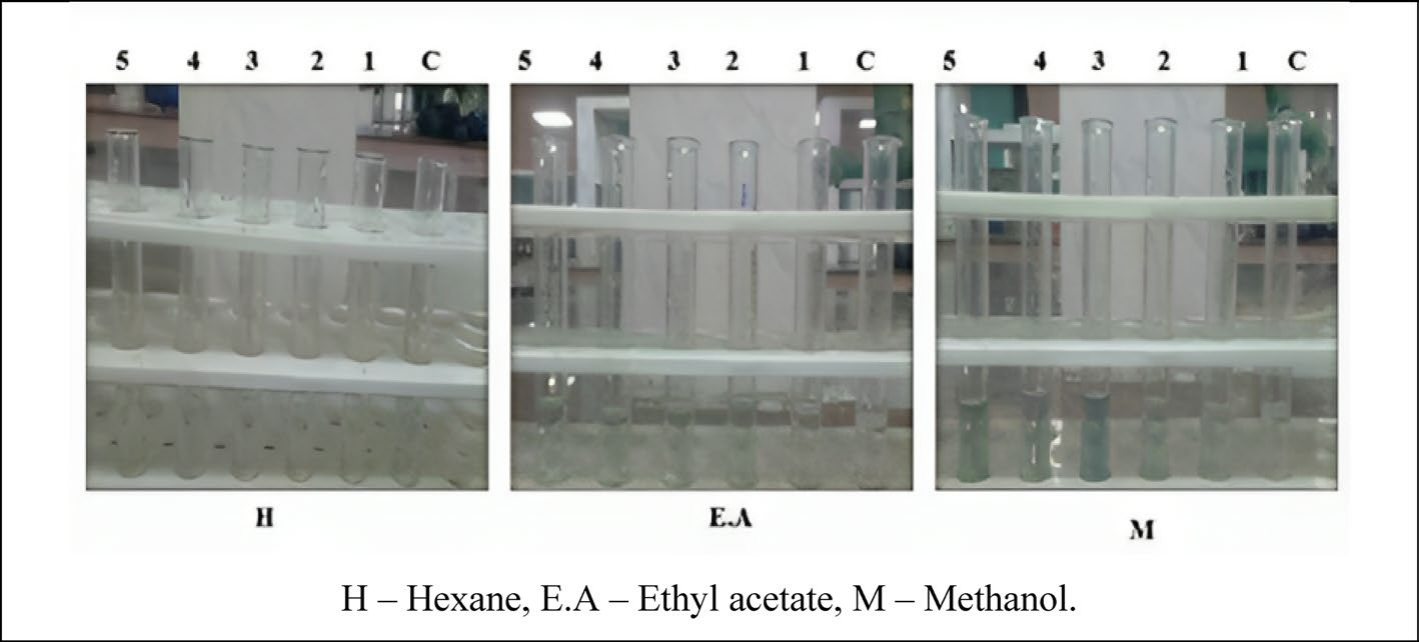
Phosphomolybdenum assay of *Artocarpus hirsutus* flower extract

**Fig. 6:**
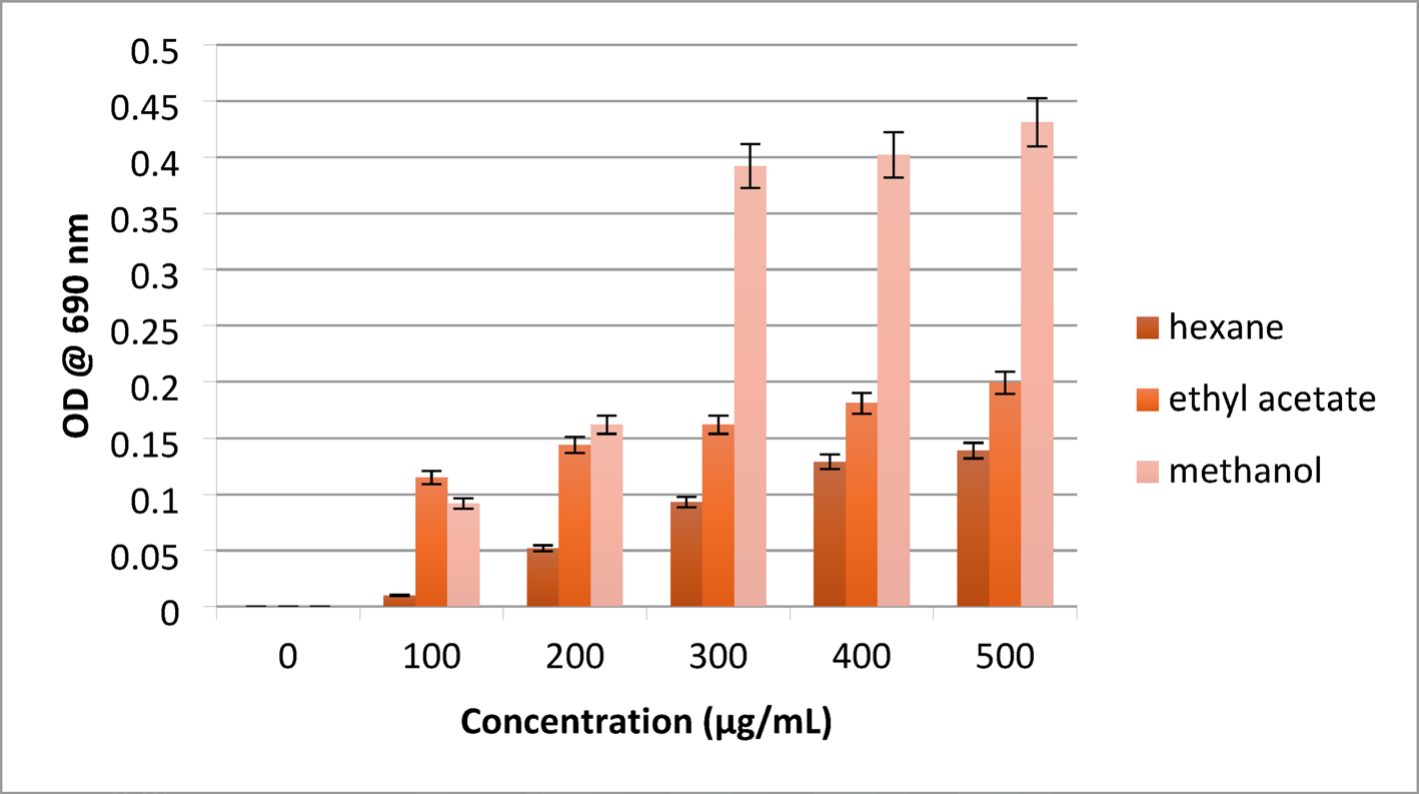
Graphical representation of Phosphomolybedium assay of *Artocarpus hirsutus* flower extract

The method of Phosphomolybdenum was used to determine the overall quantitative assessment of the antioxidant potential of the sample extracts. This technique is reduced with the help of Mo (VI) to Mo (V) by the *Artocarpus hirsutus* methanolic flower extracts and eventual development of the green phosphate/Mo (V) complex under an acidic pH condition. The intensity of the extracts at 695 nm exhibited remarkable values. The three extracts of *Artocarpus hirsutus* had been used to evaluate their antioxidant ability by developing a green Phosphomolybdenum complex. This result indicate that the extract of methanol would be a more active compound in the reduction of the Phosphomolybdenum complex compared to extracts of hexane and ethyl acetate as shown in Figs. 5 and 6. During this analysis, the sample solution transitions from its initial yellow due to varying shades of green and blue, that is determined by the reducing capacity of every biological compound. Antioxidants such as ascorbic acid, other phenolic acids, carotenoids and alpha-tocopherol are typically detected by Phosphomolybdenum [52].

#### Reducing power assay

**Fig. 7:**
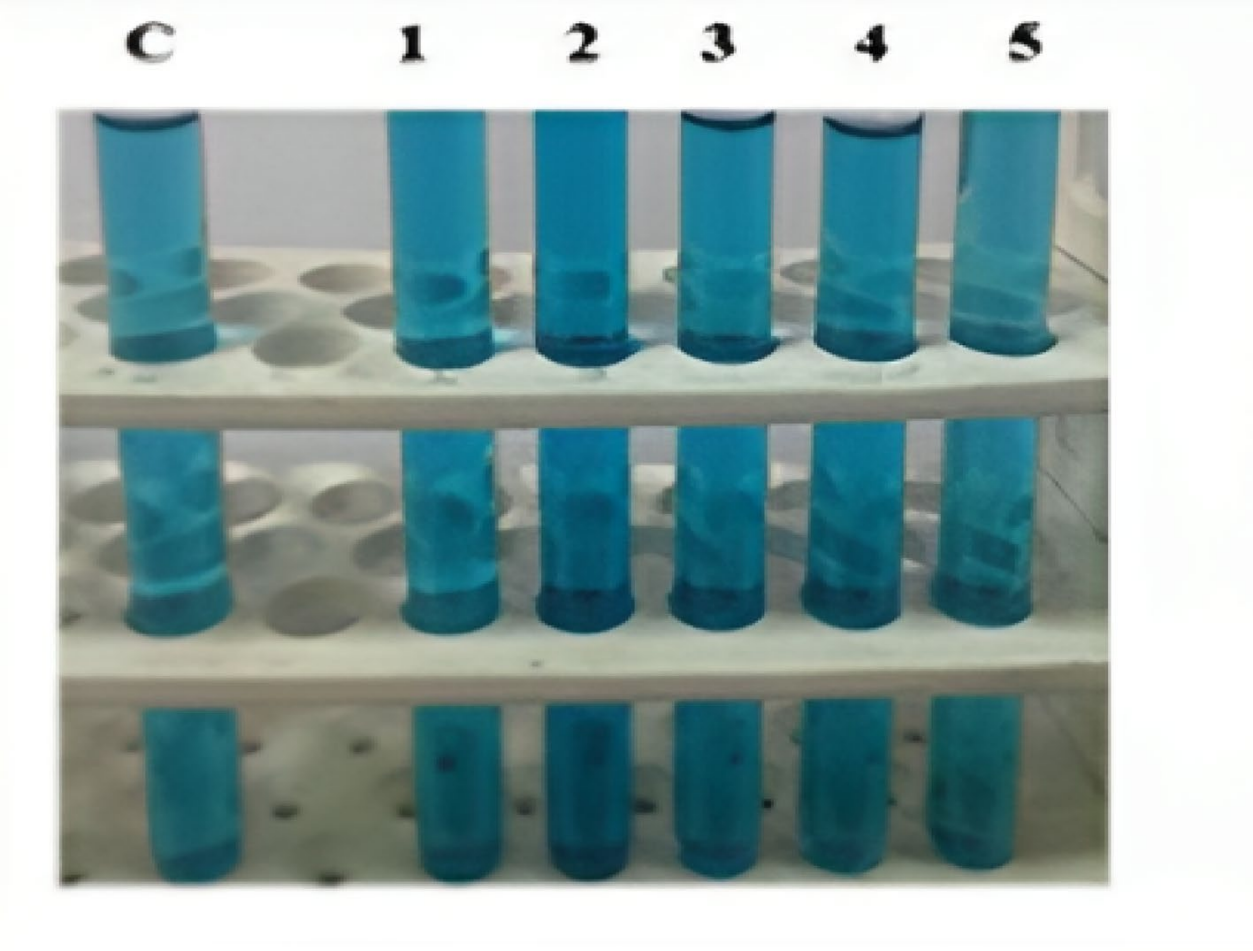
Reducing power assay of *Artocarpus hirsutus* flower extract

**Fig. 8:**
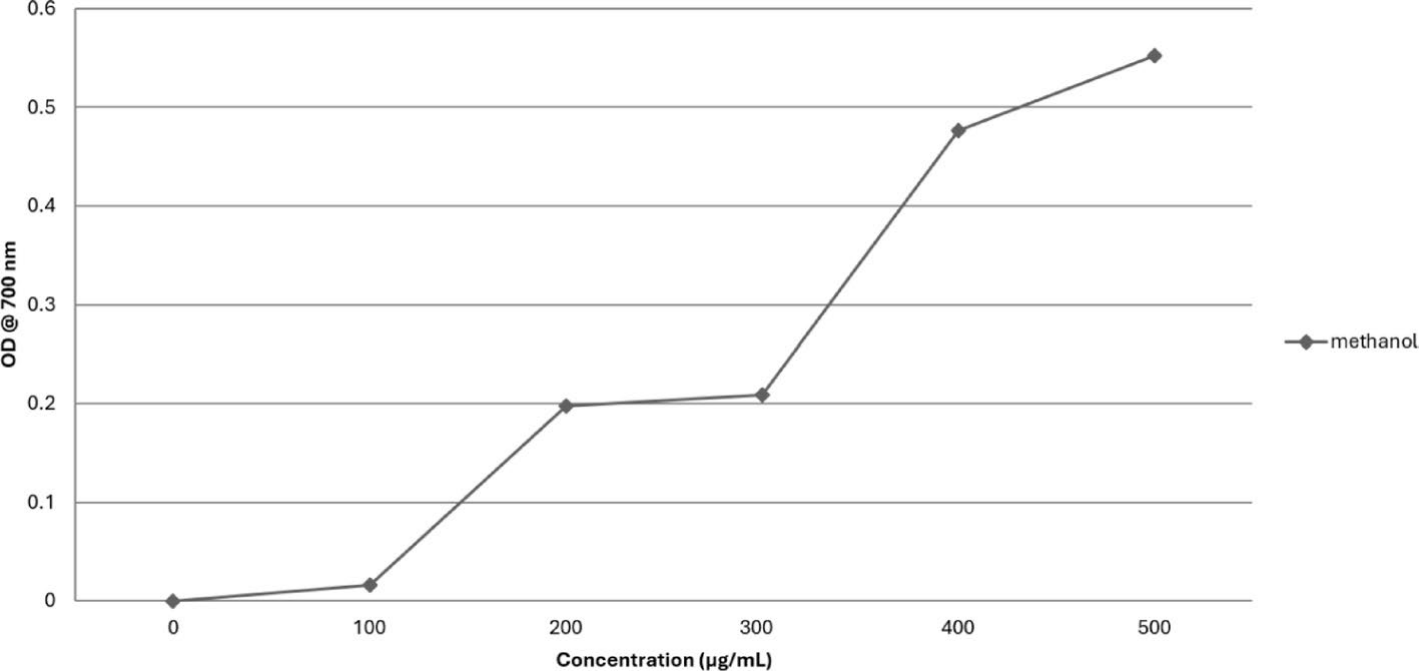
Absorbance of methanolic extract of *Artocarpus hirsutus* flower at 700 nm

The inclusion of reducers prompts the transformation of the Fe^3+^/ferricyanide complex into the ferrous form, which is employed in this procedure. The concentration of the Fe3 + ion can be calculated by measuring at 700 nm the formation of Pearl's Prussian blue [55]. The antioxidant capability of *Artocarpus hirsutus* methanol extracts as a result of their increased concentration are shown in Figs. 7 and 8. Decreasing ability can act as that of a key factor for their prominent reducing power of the compounds. Additional investigation could engage to classify the specific biological compounds which assist in power reduction. Even, as methanolic extracts showed a high antioxidant potential, further studies were conducted using methanolic extract. Reducing power assay is used to assess the oxidative impact of any product in the reaction medium based on its reduced strength. Reducing the effect of bioactive compounds has been reported to be consistent with antioxidant efficacy. Ferric power reduction of the methanolic flower extracts of *Artocarpus hirsutus* indicates that the extracts contain compounds that have a high affinity to ferrous ions, which are scavenged by redox reactions [56].

#### Lipid peroxidation assay

**Fig. 9:**
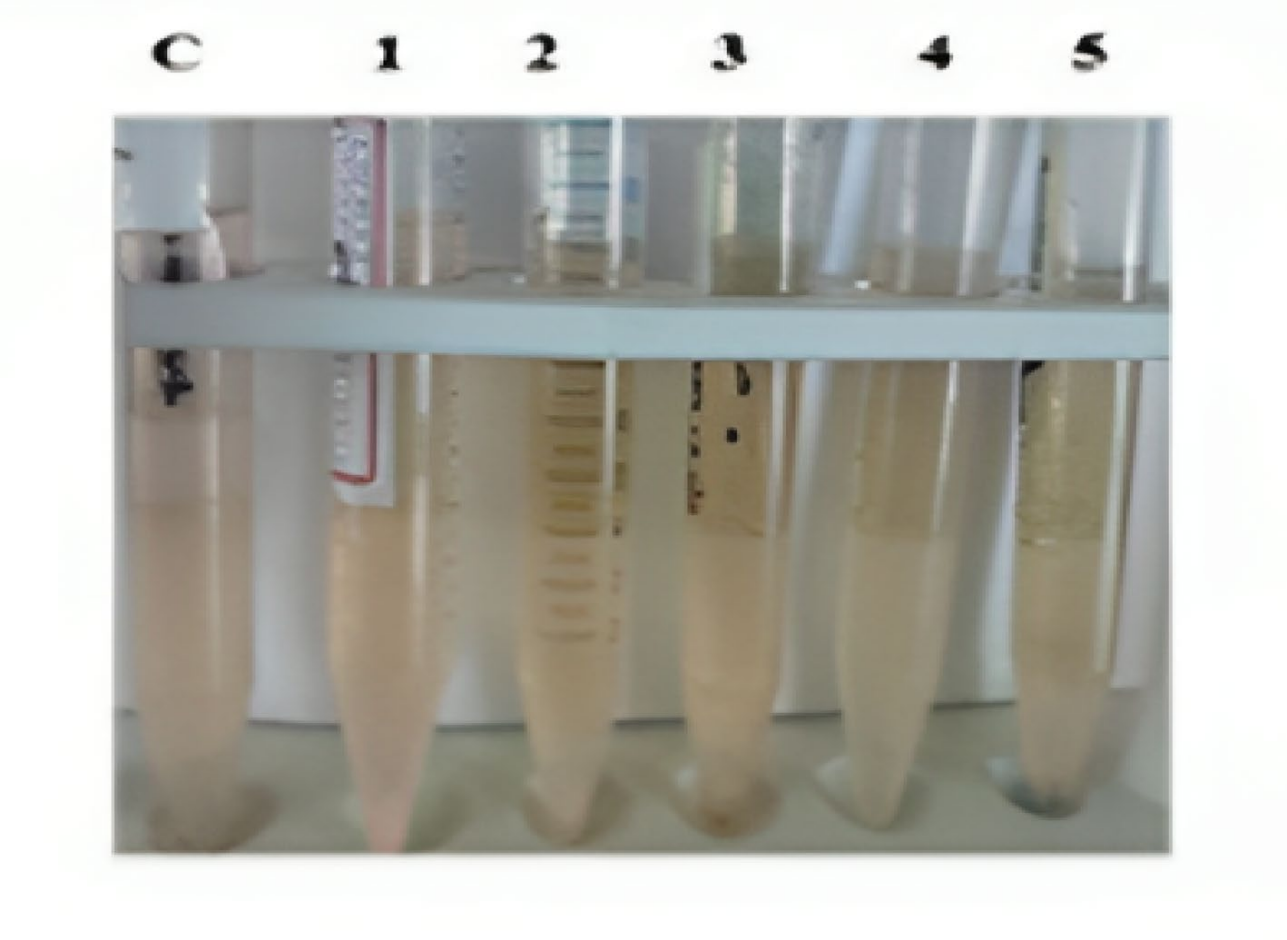
Lipid peroxidation of *Artocarpus hirsutus* flower extract

**Fig. 10:**
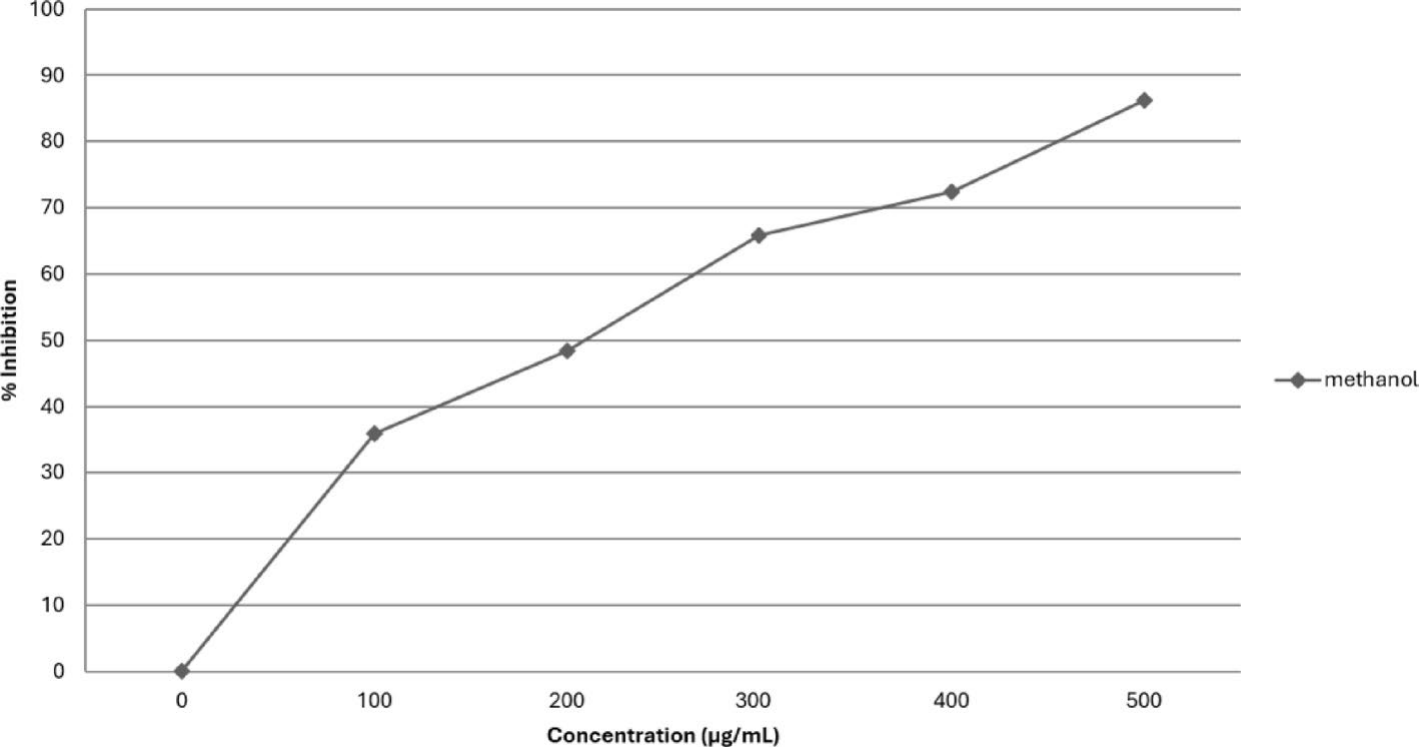
% Inhibition of *Artocarpus hirsutus* flower in methanolic extract

Lipid peroxidation experiments using goat intestine fats undergo significant non-enzymatic oxidative damage in the presence of ferrous sulphate. Lipid peroxides (LP) are likely to be involved in many pathological conditions, like inflammatory response, autoimmune diseases, cell proliferation, and cellular death [57]. The effects of different fractions of LP and ascorbic acids present in *Artocarpus hirsutus* on non-enzyme peroxidation are determined and shown in Figs. 9 and 10. The IC_50_ of the methanol extract of *Artocarpus hirsutus* was found to be 60.25 μg/mL.

### Characterization of *A. hirsutus* silver nanoparticles

#### UV spectroscopic analysis

**Fig. 11:**
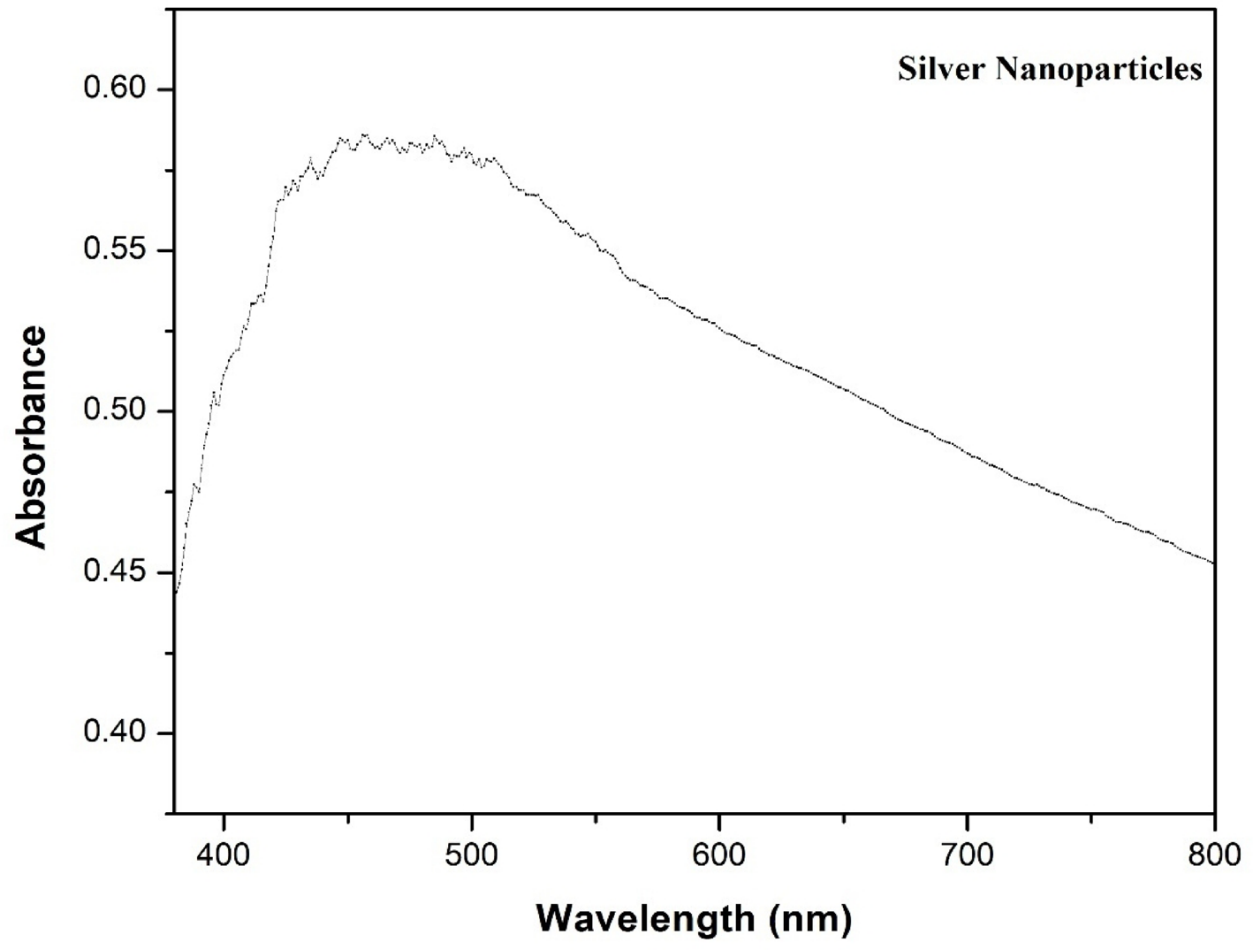
UV results of silver nanoparticles

UV Spectroscopy analysis was analyzed to determine the absorption spectrum of the synthesized nanoparticles as shown in Fig. 11. The absorption band between 400 to 500 nm confirms the presence of silver nanoparticles. The surface plasmonic resonance and (SPR) band of silver nanoparticles are based on their size, shape and also the interplanar distance. The results of the study indicate that the particles are of nano origin [58].

#### Fourier transform infrared spectroscopic analysis

**Fig. 12:**
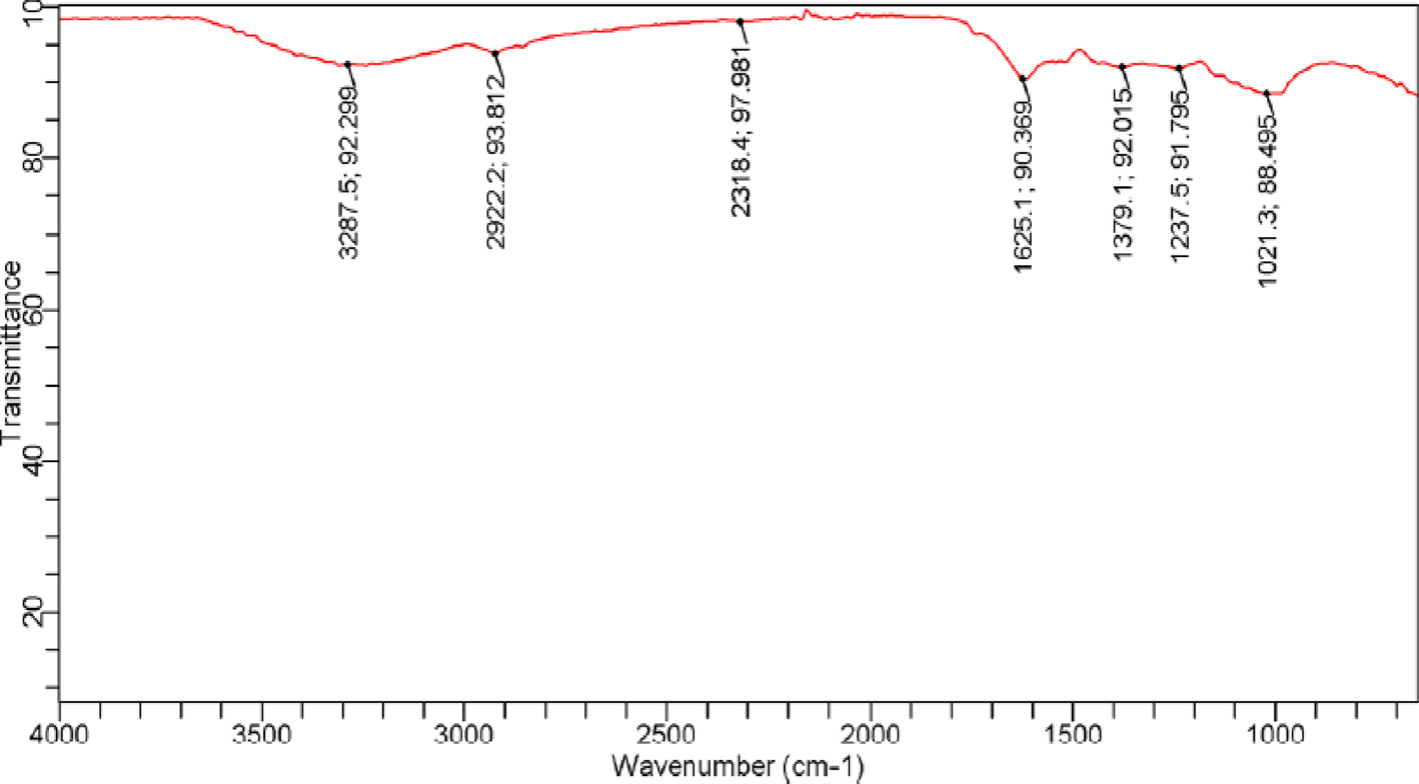
FTIR of Silver nanoparticles

The FTIR Spectrum was recorded between 4000 to 400 cm^−1^ for the synthesized silver nanoparticles as shown in the Fig. 12. The spectrum obtained at 1021 cm^-1^ is because of the P–O Stretching vibrations, 1237 cm^−1^ was due to the C–O–C stretch vibrations of the polysaccharides and the band at 1379 cm^-1^ is due to the C-O Stretching of carboxylic and amino acids. The band at 1625 cm^-1^ is because of the NH2 group stretching of amide band I. The band at 2318 cm^-1^ is due to the N–H stretch of amine and amino acids and 2922 cm^-1^ is due to O–H stretching of carboxylic acid. The stretch of 3287 cm^-1^ corresponds to the N–H stretch of amides and amino acids. Primarily derived from the protein and other phytoconstituents and polyphenolic compounds present in the plant extract, these functional groups are important in the synthesis process. These biomolecules will help to reduce the Ag⁺ to Ag⁰ (metallic silver) and cap and stabilise the nanoparticles to limit their aggregation. This confirms that the plant extract acts as a reducing and stabilising agent during the green synthesis process. This IR spectroscopic analysis confirmed the proteins having a strong binding affinity that suggest the formation of silver nanoparticles and also the plant extract that acted as the capping and stabilizing agent [39].

#### Scanning electron microscope (SEM)

**Fig. 13:**
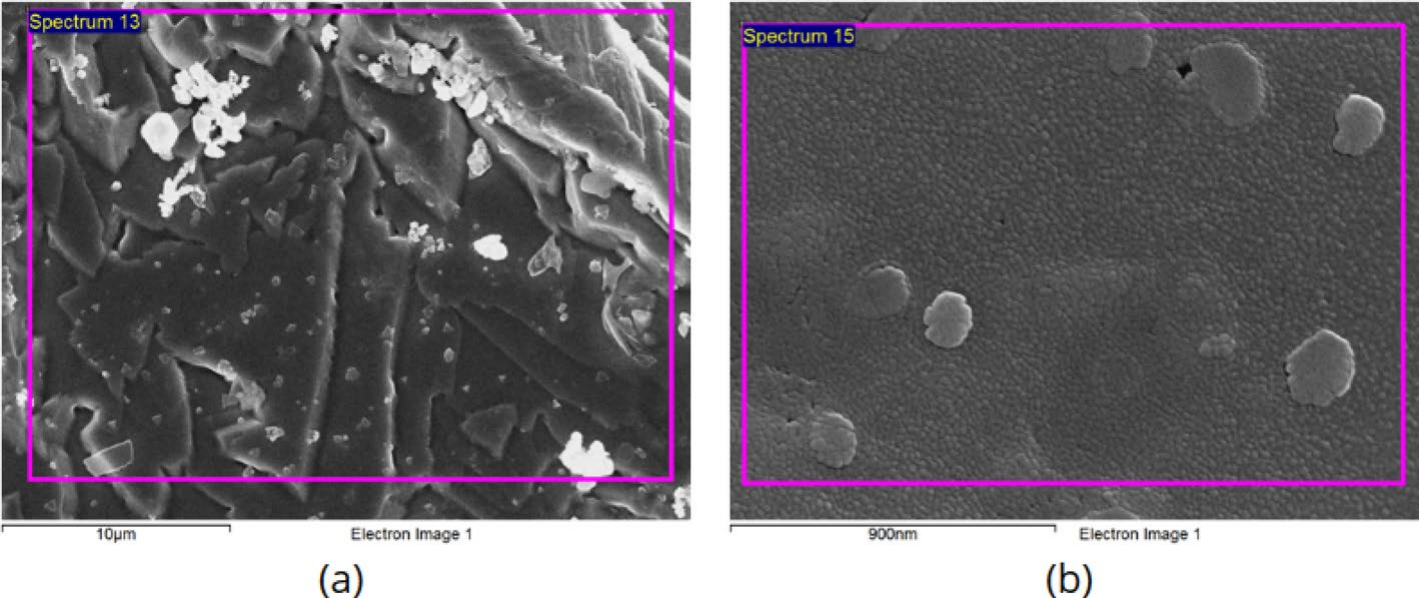
SEM image of AgNPs synthesized using *A. hirsutus* flower extract at **a** 10 µm electron image, and **b** 900 nm electron image

A graphical depiction that visually illustrates the dimension and structure of the fabricated AgNPs are shown in Fig. 13. This image shows that the AgNPs provides a variety of shapes and sizes, lacking uniformity. The micrographs display various geometric forms with predominant spherical structure, including truncated hexagonal, cylindrical, triangular, and prismatic shapes. The typical grain size of these AgNPs falls within the range of 10 to 50 nm. Due to their substantial surface area, the particles tend to cluster together slightly, giving rise to medium-sized particles [59].

#### Energy dispersive absorption (EDAX) analysis

**Fig. 14:**
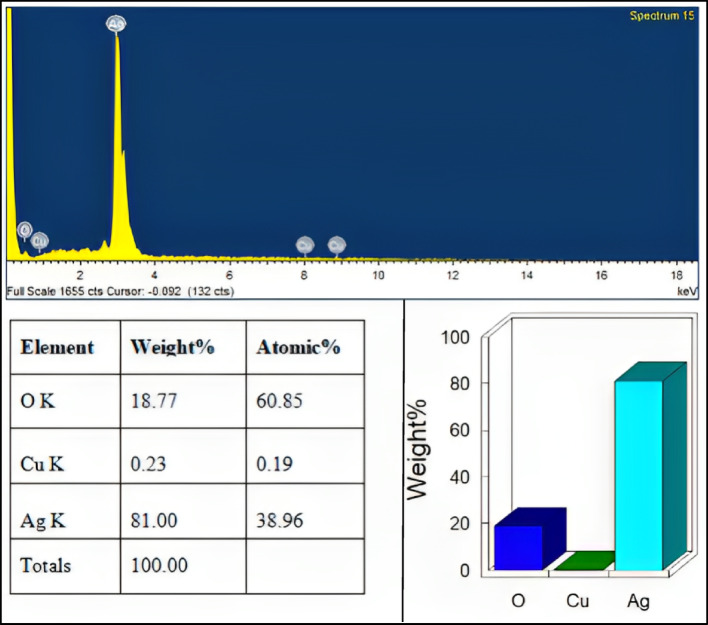
EDAX Spectrum of *A. hirsutus* silver (AgNPs) nanoparticles

Elemental composition analyses for the produced AgNPs were conducted by utilizing energy dispersive absorption (EDAX) technique, and the results are presented in Fig. 14. In this analysis, it was confirmed that silver (Ag) was indeed present in the final product, verifying the presence of the essential element. The study demonstrated that the nanostructures produced by *A. hirsutus* generated a distinct peak at 3.2 keV, which exclusively corresponds to silver nanoparticles [60]. The EDAX analysis further substantiates the elemental composition of the AgNPs synthesized from the flower extract of *A. hirsutus*, and these results have been tabulated. A very minor copper signal (0.23%) was also detected, which may arise from the instrument or sample grid rather than from the synthesized nanoparticles themselves. Overall, the EDAX results confirm the elemental composition and successful synthesis of silver nanoparticles.

#### X-ray diffraction (XRD)

**Fig. 15:**
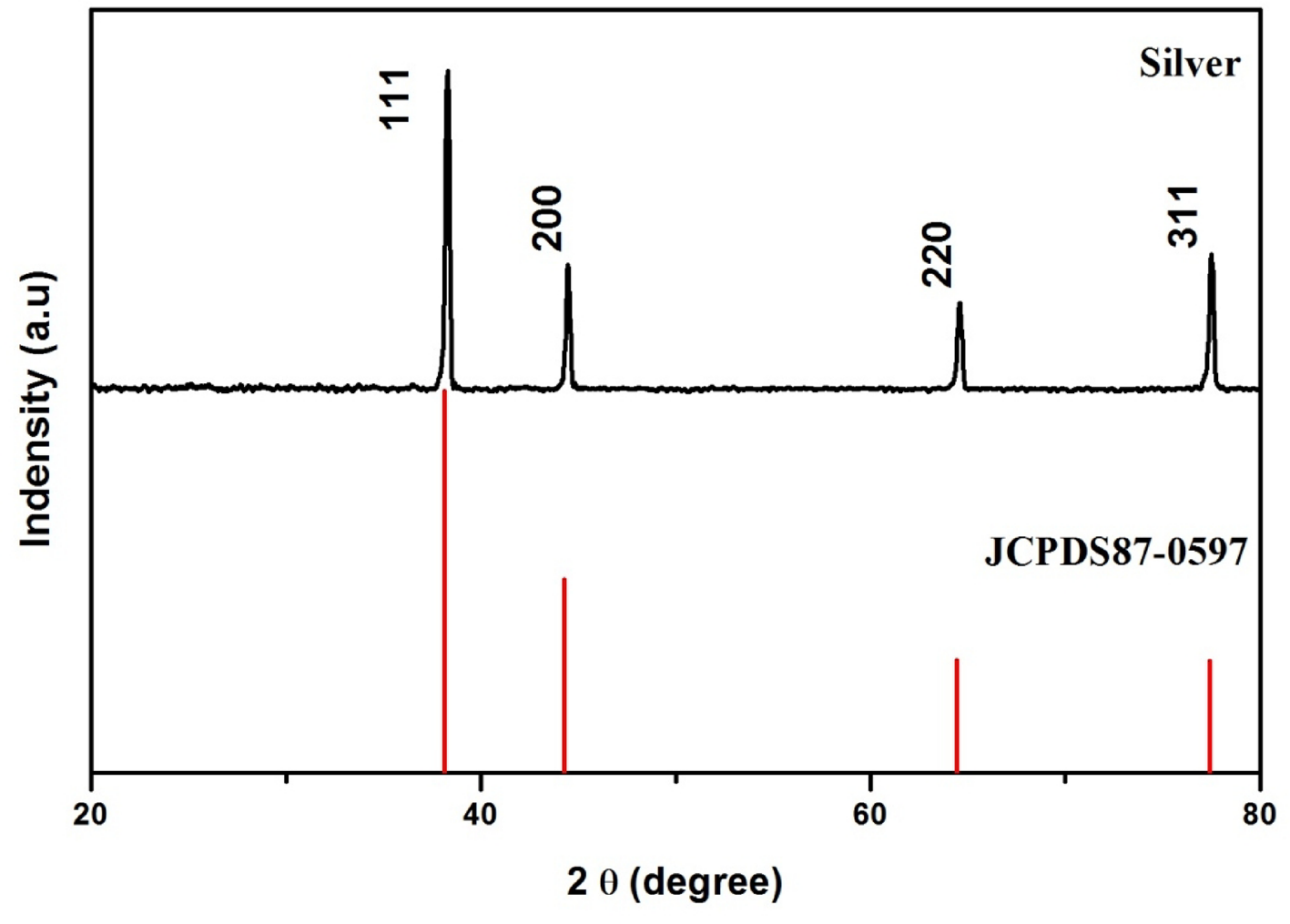
X-ray diffraction (XRD) analysis of *A. hirsutus silver* nanoparticles

Under optimized physicochemical circumstances, *A. hirsutus* methanolic flower extract was utilized for the production of silver nanoparticles (AgNPs) and examined to have a crystalline structure through XRD analysis. The XRD pattern for the AgNPs obtained from *A. hirsutus*, revealing the presence of four distinct crystallographic peaks at 2θ values of 38.99°, 44.29°, 64.59°, and 78.62° was depicted in Fig. 15. Upon analysis, these XRD patterns were determined to be in alignment with the indexed Face Center Cubic (FCC) structure, specifically corresponding to the (111), (200), (220), and (311) diffraction planes of AgNPs. This unequivocally verifies the AgNPs produced with the extract from *A. hirsutus* flowers that exhibit a crystalline structure. This study highlights the successful synthesis of nanoparticles utilizing *A. hirsutus* flower as both a reducing and capping agent. It also underscores the essential role of XRD analysis in confirming the crystallographic characteristics of the fabricated nanoparticles. The identification of specific diffraction peaks and their corresponding 2θ values furnishes quantitative data to support the comprehensive characterization of the produced nanoparticles [61].

### Cytotoxicity study

**Fig. 16:**
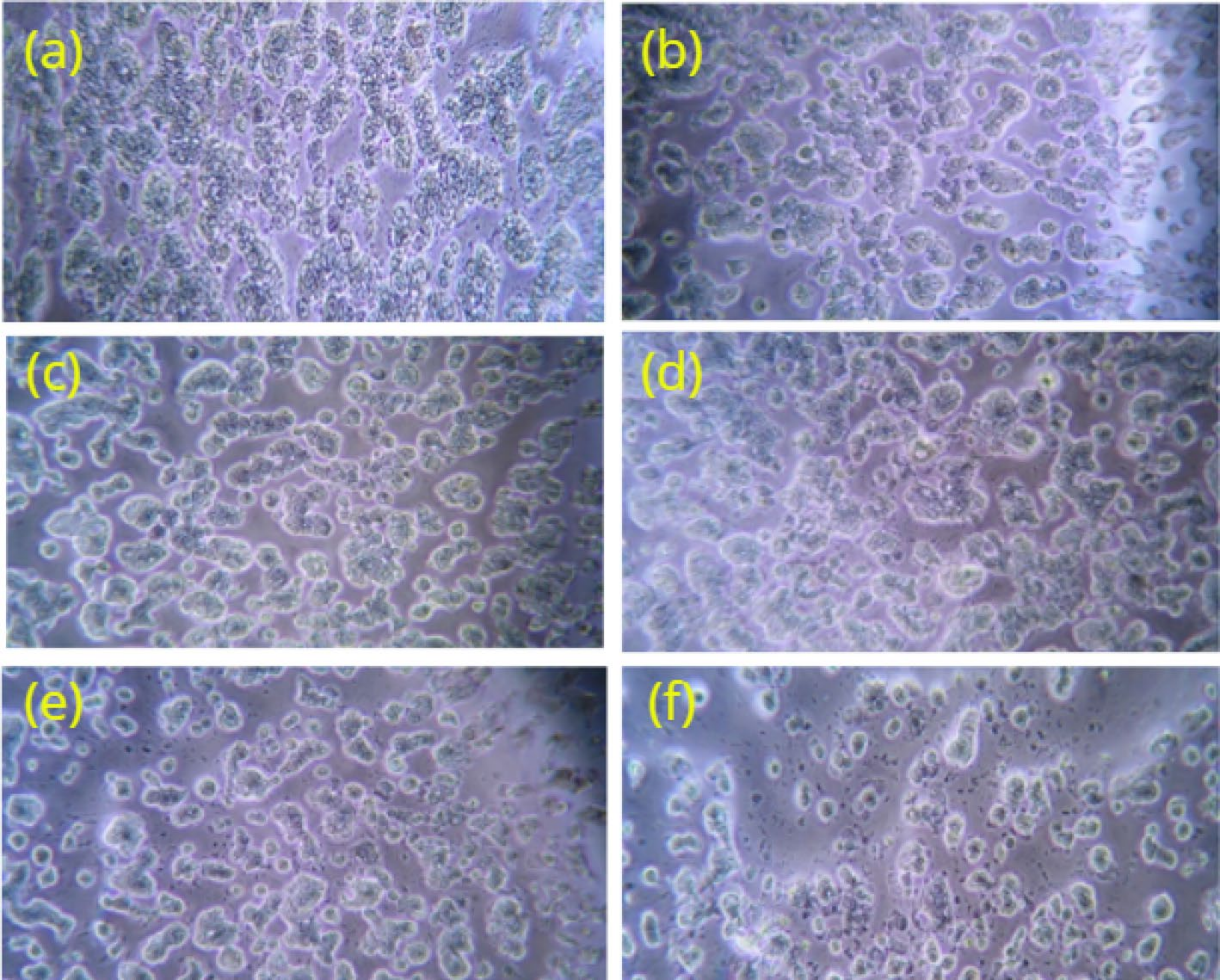
Morphological analysis of *A. hirsutus* AgNPs treated against HT29 colon cancer cells in **a** Control **b** 25 µg/ml **c** 50 µg/ml **d** 100 µg/ml **e** 250 µg/ml **f** 500 µg/ml

**Fig. 17:**
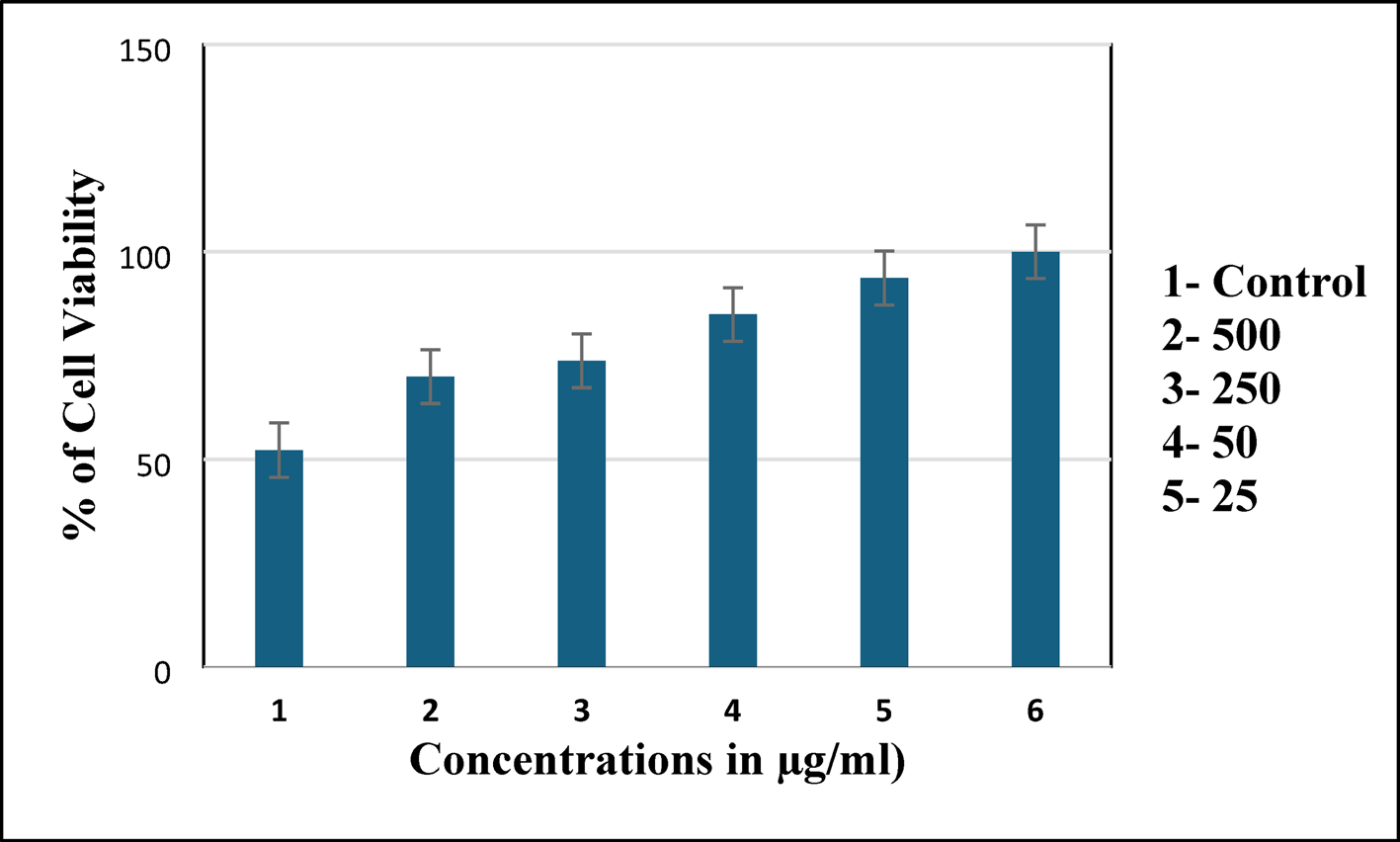
Cytotoxic efficacy of fabricated silver (AgNPs) nanoparticles on HT29 colorectal adenocarcinoma cells

From this study, we infer that the synthesized silver (AgNPs) nanoparticles by the cytotoxicity analysis on HT29 cells. This revealed that as the AgNPs concentration increased, there was a notable reduction in cell viability. The flower extracts of *Artocarpus hirsutus* silver nanoparticles against HT-29 colorectal adenocarcinoma cell line in no treatment (control) as well as the treatment with concentration of 25 to 500 µg/ml are depicted in Fig. 16. IC_50_ value obtained for silver (AgNPs) nanoparticles was 446 µg/ml and this study investigates the cell viability of were produced using methanol extract derived from the blossoms of *A. hirsutus*. From our results, it is clear that smaller nanoparticles are capable of easily infiltrating the tumor matrix, leading to the destruction of cancer cells [62]. The relatively high IC₅₀ value (~446 µg/mL) indicates moderate potency and highlights the need for further optimization. From the Fig. 17s , the IC_50_ values of AgNPs shows potentially more cytotoxic against human colorectal adenocarcinoma, such cytotoxic efficacy of the nanoparticles that were created may be attributed to the way in which biological molecules on their surfaces interact [63]. Thus, biologically produced AgNPs proves to be cytotoxic against HT-29 colorectal carcinoma cell lines (Fig. 16).

### ROS measurement by DCFDA staining

**Fig. 18:**
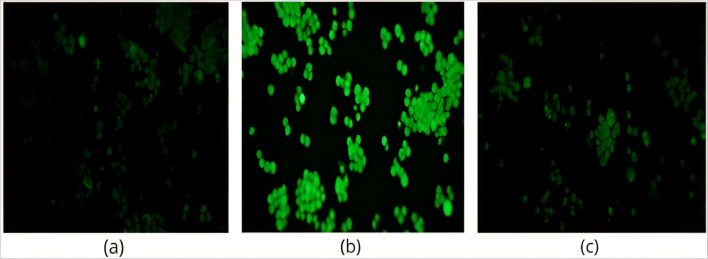
Production of ROS in synthesized nanocomposites at their IC_50_ using H_2_DCFDA staining in **a** Control **b** Positive control and (C) AgNPs

This research aimed to assess the production of ROS in HT-29 colorectal carcinoma cell lines following exposure to AgNPs. The highest level of green fluorescence was detected in the cells that had been exposed with AgNPs as shown in Fig. 18. The fluorescence observed was a result of the generation of ROS within cells that have been treated. These ROS ions are very reactive and play a vital role in cellular communication, being produced internally by the cell. Elevated ROS levels stimulate the release of TNF-α, an inflammatory biomarker, which in turn triggers the activation of nuclear factor-κB and c-Jun N-terminal kinase pathways, ultimately leading to both programmed as well as necrotic cell death. Reactive oxygen species (ROS), which are free radicals, have a significant impact on causing apoptosis and cell damage within living organisms [64]. The heightened cell death observed in cells exposed to AgNPs was a result of the nanoparticle’s dimensions, morphology, and their level of potency, which collectively led to an elevation in ROS levels [65].

### DNA fragmentation assay

**Fig. 19:**
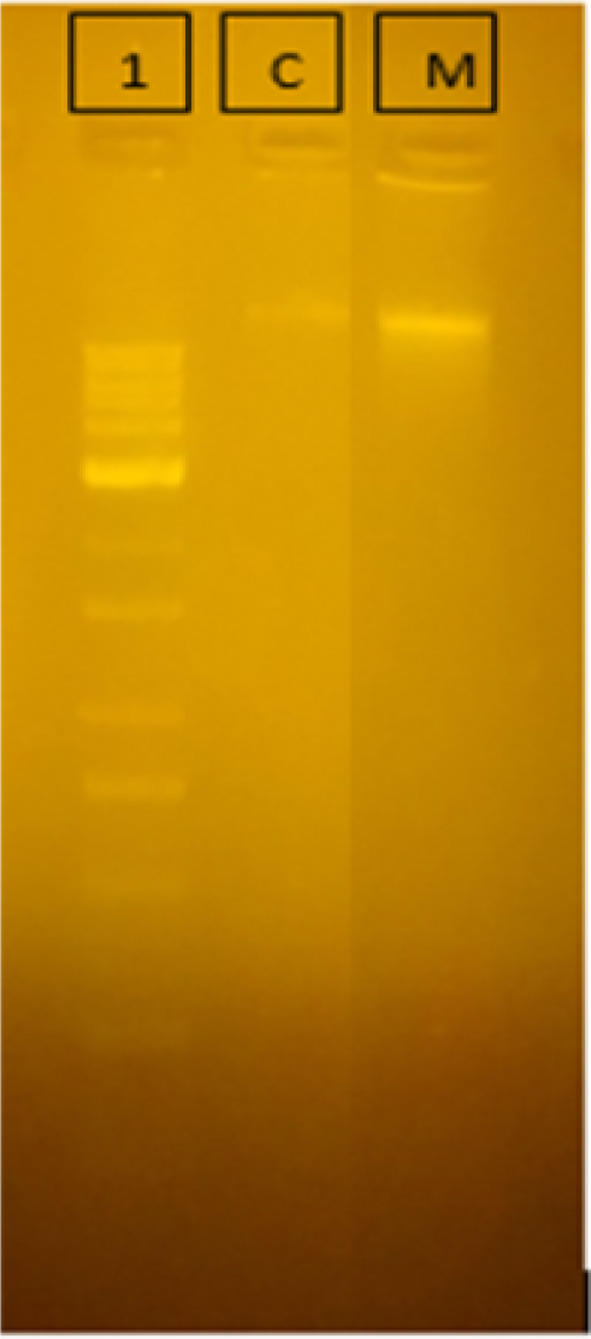
Apoptotic DNA laddering Analysis of AgNPs. 1–DNA sample exposed to silver nanoparticles M–1 kb; C–Control HT29; DNA-ladder (GeneDirex)

This study focused on examining the ability of synthesized AgNPs to trigger apoptosis by conducting a DNA laddering analysis and to investigate whether nanoparticles produced through biological means induce cell death through apoptosis using an agarose gel. The outcomes revealed that when using a flower extract from *Artocarpus hirsutus*, the synthesized AgNPs induces DNA ladder formation resulting in a 1 kb ladder pattern in HT-29 colon cancer cells, exhibiting distinctive characteristics on programmed cell death. The intensity of the laddered DNA breaks was notably higher in the cells treated with *Artocarpus hirsutus*, as observed through agarose gel electrophoresis, in comparison to the control cells, which were left untreated and displayed no such DNA laddering analysis. The DNA fragments observed in the tested samples showed a programmed cell death and revealed when AgNPs were applied to HT-29 colorectal carcinoma cell lines, there were significant double stranded ladder in the DNA, resulting in a ladder-like pattern. In contrast, control HT29 colorectal carcinoma cell lines, which were supplemented with 10% serum, experienced minimal DNA breakage. To determine the molecular weight of the cleaved DNA fragments, a 1 kb ladder was utilized. The DNA laddering analysis results provided strong evidence that HT29 colorectal carcinoma cells treated with silver nanoparticles exhibited extensive double-stranded breaks, displaying a ladder-like pattern as shown in Fig. 19. The heightened level of fragmentation observed provides evidence that the synthesized AgNPs have indeed triggered apoptosis in HT29 colorectal carcinoma cells [66, 67].

### Assessment of flow cytometry

**Fig. 20:**
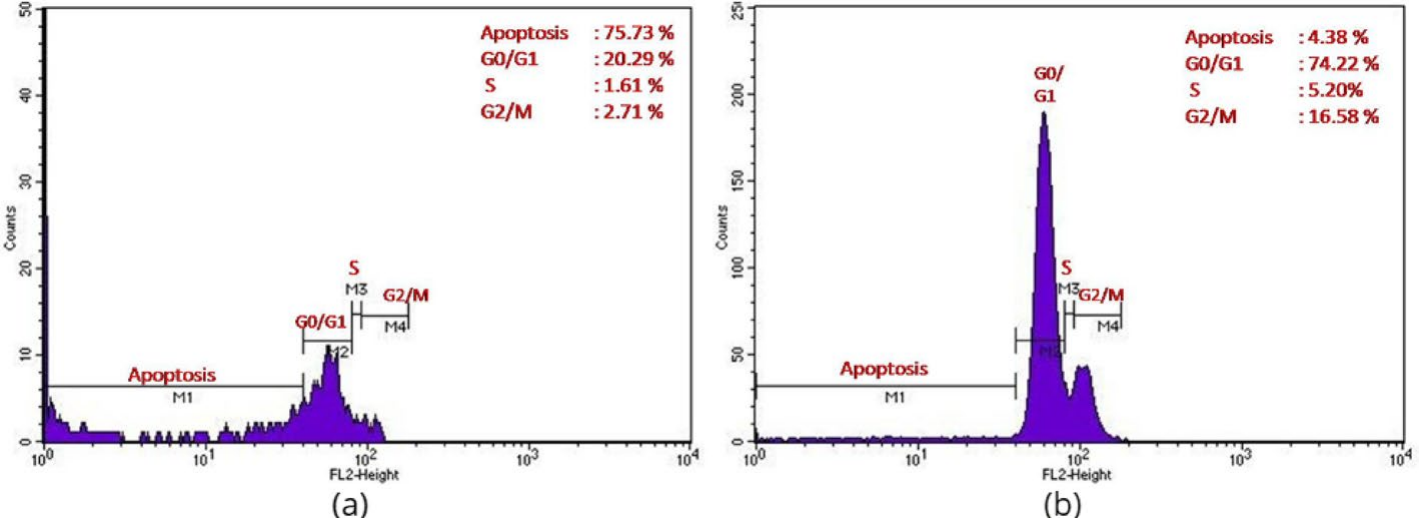
Graphical representations of flow cytometry representing AgNPs against HT-29 colorectal adenocarcinoma cells with IC_50_ concentrations in Control (**a**), and AgNPs (**b**)

The cell cycle may be a complex and convoluted process due to the various signals that cells receive to regulate their growth, which are examined at multiple checkpoints [68]. The efficacy of *Artocarpus hirsutus* fabricated silver (AgNPs) nanocomposites at their IC_50_ concentrations were investigated on the cellular development of HT-29 colorectal adenocarcinoma cells by flow cytometry as illustrated in Fig. 20.

**Fig. 21:**
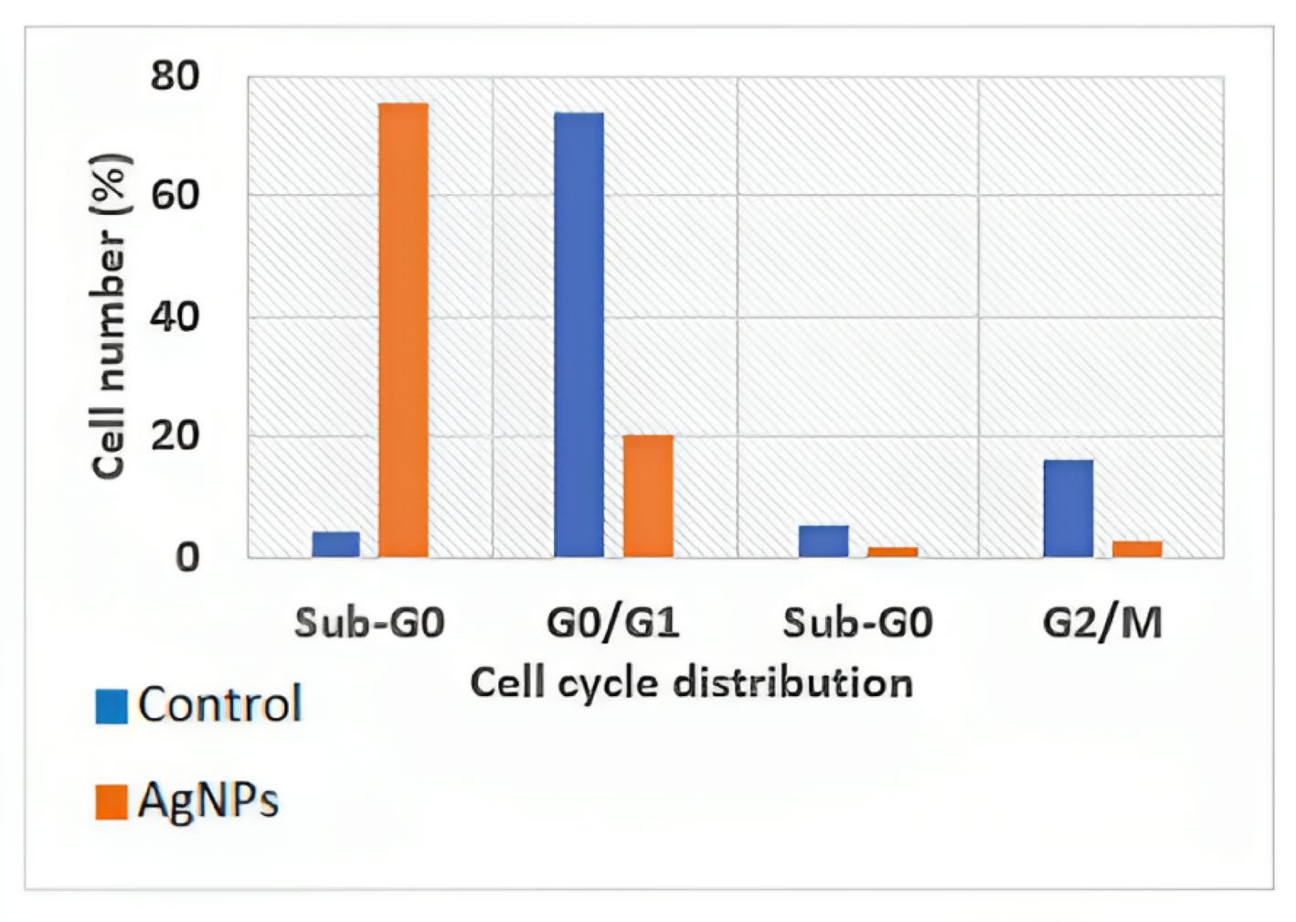
A bar chart illustrating various phases of the cell cycle in the samples against *A. hirsutus* silver (AgNPs) nanoparticles

The silver (AgNPs) nanoparticles produced from flower extract of *A. hirsutus* showed substantial arresting at the stage of G1 phase during cell cycle while simultaneously reducing the number of cells in the stage of S phase as well as G2/M phase. They were examined at the IC_50_ concentration of the green synthesized nanoparticles. Analyzing the graphical representations, it was evident that these nanoparticles induced an arrest in the cell cycle of colorectal carcinoma cell lines, and an efficient programmed cell death was illustrated in Fig. 21. This effectiveness was more pronounced in the silver nanoparticle treated samples. Similar observations were noticed in the study where silver nanoparticles resulted in effective apoptotic cell death [68, 69]. Therefore, our findings strongly suggest that the use of *A. hirsutus*-driven silver nanoparticles (AgNPs) triggers an apoptotic pathway in HT-29 colorectal adenocarcinoma cells.

### Gene expression analysis

**Fig. 22:**
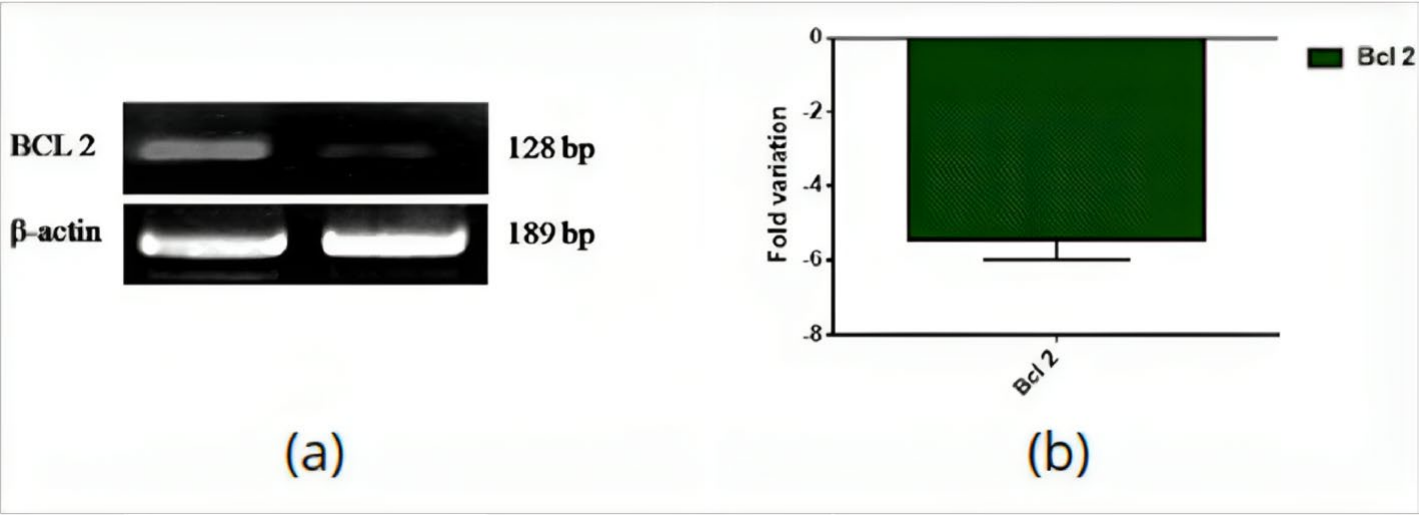
Anti-apoptotic gene expression analysis of AgNPs. **a** Control **b** Influence of AgNPs on the regulation of *Bcl2* mRNA levels in HT29 cells

The study aimed to confirm the gene expression of the anti-apoptotic Bcl2 in HT-29 colorectal carcinoma cells treated with AgNPs using qRT-PCR. The research findings indicate the alteration in fold change and PCR investigation of the AgNPs-treated cell lines are shown in Fig. 22. Our findings clearly show a substantial reduction in the down regulation of the Bcl2 gene, which was particularly prominent in the cell lines exposed to produce AgNPs compared to the control group. To ensure the accuracy of our RT-PCR results, we used β-actin as a standardized internal control gene. The down regulation of Bcl2 is associated with the initiation of apoptosis, leading to the destruction of adenocarcinoma cells. Since mitochondria are central to the apoptotic signaling process, any disruption in their integrity can trigger apoptotic cell death. Our research suggests that the production of reactive oxygen species (ROS) is primarily induced at the mitochondria, primarily due to the involvement of AgNPs. These findings suggest that HT29-colorectal carcinoma cells exhibit heightened sensitivity to induce apoptosis when subjected to AgNPs. Nanoparticles can potentially trigger apoptosis in carcinoma cells through two main mechanisms. Firstly, by generating reactive oxygen species (ROS), and secondly, by modulating specific proteins within the carcinoma cells, ultimately driving them towards cell death [70]. Previous reports have exposed the cytotoxic and apoptotic properties of AgNPs in different cell types [71, 72].

## Discussion and conclusion

In this research, a safe and efficient method for synthesizing AgNPs using a methanol flower extract of *Artocarpus hirsutus* was described. Compared to conventional chemical methods, the present approach offers a simplified synthesis route by eliminating the need for external reducing and stabilizing agents, thereby enhancing its sustainability and applicability in green nanotechnology [73]. Various analytical techniques, including UV–Vis, SEM, FTIR, and EDAX spectroscopy, were employed to comprehensively analyze the environmentally friendly synthesized nanoparticles. The SEM images depict a size distribution of nanoparticles ranging from 10 to 50 nm, displaying both potential spherical and irregular geometries. According to the FTIR findings, it was proposed that the phytoconstituents found in *Artocarpus hirsutus* played a crucial role in reducing silver ions and capping the resulting AgNPs. Furthermore, EDAX analysis patterns confirmed the elemental composition of the AgNPs, with copper being the dominant element at 3.2 keV. When these nanoparticles were tested for in vitro cytotoxicity effects on HT29 colon cancer cell lines, a maximum efficacy of 74.8% was observed at the highest concentratio (500 µg/ml µg/L). The synthesized AgNPs exhibited moderate cytotoxic activity against HT-29 cells. The observed IC₅₀ value may vary from previous reports due to differences in the plant source and synthesis conditions. Further optimization may enhance the anticancer efficacy of the synthesized AgNPs. This assessment was conducted using various analyses, including the MTT assay, gene expression profiling, DNA fragmentation, and staining assays. The present findings are consistent with previous reports demonstrating that biogenically synthesized silver nanoparticles possess multiple biological activities, including anticancer, anti-inflammatory, and antimicrobial effects. These activities are largely attributed to oxidative stress induction and apoptosis-mediated mechanisms, supporting the observed cytotoxic effects in cancer cells [74]. The study demonstrated that the environmentally friendly synthesized AgNPs exhibited potential inhibiting effects on the development of human colorectal adenocarcinoma HT-29 cells. The observed dose-dependent cytotoxicity in HT-29 cells is in line with previous studies reporting that increasing concentrations of silver nanoparticles induce significant cellular damage, including chromosomal aberrations and reduced cell proliferation. Such findings highlight the potential of AgNPs to disrupt cellular integrity and contribute to cytotoxic effects through concentration-dependent mechanisms [75]. Additionally, it appeared that higher concentrations of AgNPs from *A. hirsutus* induced more pronounced morphological changes, indicating that apoptosis occurred in a concentration-dependent manner. All experiments were performed in triplicate, and the results are presented as average values of three independent experiments. Statistical analysis was carried out using GraphPad Prism 9 and IBM SPSS software. Statistical significance was evaluated using one-way ANOVA with a threshold of *p* < 0.05, where applicable. The IC₅₀/LC₅₀ values were determined using ED50PLUS software. The observed increase in ROS levels together with the downregulation of Bcl-2 expression suggests the possible involvement of apoptosis-related pathways in AgNP-mediated cytotoxicity. However, these findings do not conclusively establish the underlying molecular mechanism, and further studies are required to confirm the precise apoptotic pathways involved. Further studies involving detailed molecular pathway analysis are required to confirm the apoptotic pathways involved. These findings suggest apoptosis-inducing effects of AgNPs in HT-29 cells under in vitro conditions. The present study did not include DLS, zeta potential, and TEM analyses. Therefore, although SEM and EDAX confirmed the morphology and elemental composition of the synthesized AgNPs, further physicochemical characterization is required and will be considered in future studies. Although the synthesized AgNPs demonstrated significant cytotoxic effects against HT-29 colon cancer cells, their selectivity toward cancer cells compared with normal healthy cells could not be determined. Therefore, further investigations using normal colon epithelial cells and other non-cancerous cell models are necessary to evaluate the safety profile and therapeutic selectivity of the synthesized nanoparticles. The present study is limited to a single cancer cell line (HT-29), and no comparison with normal cell lines was performed. Therefore, the selectivity and therapeutic safety of the synthesized AgNPs cannot be established at this stage.

This study underscores the potential of AgNPs, produced using a methanol flower extract of *A. hirsutus*, not only in terms of their minimal environmental impact but also as potentially anticancer agents. Therefore, the current findings should be considered as an initial step toward exploring the biological activity of plant-mediated AgNPs rather than conclusive evidence of anticancer efficacy. In summary, AgNPs from *Artocarpus hirsutus* via green synthesis have shown both dose-dependent cytotoxic effects as well as induction of apoptotic cell death to HT-29 (human colon) cancer cell line. These possible cellular mechanisms of toxicity may be through oxidative stress via ROS and reduction in anti-apoptotic proteins such as Bcl-2. All of these findings show that the nanoparticles have the potential to have biological activity; however, all of these results were acquired in vitro and with one type of cancer cell. Therefore, more studies need to be performed using both non-cancerous cell lines, in vivo animal models and additional mechanistic studies before AgNPs can be utilized in a clinical trial for cancer. Further progress in this field requires interdisciplinary collaboration among nanotechnologists, biologists, and clinicians to overcome existing challenges and to translate nanoparticle-based systems into effective biomedical applications.

Future research should focus on comprehensive in vivo validation, advanced mechanistic investigations at the molecular level, and the development of targeted and controlled nanoparticle delivery systems. The present study did not include plant extract-alone or AgNO_3_ treated control groups, which limits the ability to distinguish the individual contributions of phytochemicals, silver ions, and the synthesized AgNPs to the observed biological effects. Furthermore, the integration of computational approaches, including molecular modeling, molecular docking, and systems-level network analysis, with experimental studies can provide deeper insights into nanoparticle cell interactions, enabling rational design and optimization of more efficient and safer nanotherapeutic platforms.

## Supplementary Information

Below is the link to the electronic supplementary material.

Supplementary Material 1

## Data Availability

Data supporting the findings of this study are available from the corresponding author upon reasonable request.
