# Supplementary material for: Green synthesized silver nanoparticles from Artocarpus hirsutus flower extract induced cytotoxicity and apoptosis in HT29 colon cancer cells
Source: Discov Nano. 2026 Aug 10;21(1):384. doi: 10.1186/s11671-026-04834-w (PMC13457538; doi:10.1186/s11671-026-04834-w)
Supplement: Supplementary file 1 [file 11671_2026_4834_MOESM1_ESM.docx]

**Uncropped Gel Images**
